# Therapeutic Interventions in Niemann–Pick Disease Type C: A Systematic Review of Clinical Outcomes and Safety

**DOI:** 10.3390/biomedicines14102402

**Published:** 2026-10-21

**Authors:** Zaina Nafees, Sara S. N. Mohammed, Grisilda Vidya Bernhardt, Suresh Kumar

**Affiliations:** 1 RAK College of Medical Sciences, RAK Medical and Health Sciences University, Ras Al Khaimah 11172, United Arab Emirates; 2 Department of Biochemistry, RAK College of Medical Sciences, RAK Medical and Health Sciences University, Ras Al Khaimah 11172, United Arab Emirates; 3 Department of Pharmacology, RAK College of Medical Sciences, RAK Medical and Health Sciences University, Ras Al Khaimah 11172, United Arab Emirates

**Keywords:** Niemann–Pick disease type C, NPC, miglustat, arimoclomol, N-acetyl-L-leucine, hydroxypropyl-β-cyclodextrin, efavirenz, nizubaglustat, systematic review, lysosomal storage disorder

## Abstract

**Background**: Niemann–Pick disease type C (NPC) is a rare, progressive lysosomal storage disorder characterized by impaired intracellular lipid trafficking, leading to systemic, neurological, and psychiatric manifestations. Although supportive care was historically the main treatment approach, several disease-modifying and symptomatic therapies have recently emerged. This systematic review aimed to evaluate the efficacy and safety of current therapeutic strategies for NPC. **Methods**: A systematic literature search was conducted in PubMed, Scopus, and ClinicalTrials.gov from inception to 1 August 2026. Eligible studies included randomized controlled trials and interventional clinical studies involving patients with NPC of any age or sex. Interventions included miglustat, arimoclomol, N-acetyl-L-leucine (NALL), hydroxypropyl-β-cyclodextrin (HPβCD), vorinostat, N-acetylcysteine (NAC), efavirenz, nizubaglustat and other therapeutic strategies. Outcomes included neurological progression, clinical severity scores, ataxia measures, quality of life, biomarkers, cognition, and adverse events. Due to heterogeneity in study designs and outcome measures, findings were synthesized narratively. **Results**: Twenty-four studies representing approximately 475 participants, after removal of documented cohort overlap, were included. Miglustat showed evidence of neurological stabilization, particularly when initiated early, with mainly gastrointestinal adverse effects. Arimoclomol demonstrated evidence of slowing neurological progression and was generally well tolerated, although longer-term safety and effectiveness data remain necessary. NALL improved ataxia-related symptoms and was generally well tolerated. HPβCD demonstrated biological target engagement, including changes in cholesterol-related biomarkers, but clinical findings were mixed: uncontrolled intrathecal and intravenous studies suggested stabilization or improvement, whereas controlled evidence was less supportive. Hearing impairment remained an important safety concern. Evidence for vorinostat, NAC, efavirenz, and nizubaglustat was limited by small samples and early-phase or uncontrolled designs. **Conclusions**: The available evidence most consistently supports miglustat, arimoclomol, and NALL for stabilizing neurological progression or improving functional outcomes in NPC. However, differences in study design, outcome measures, and follow-up preclude direct comparison between therapies. HPβCD remains investigational because its biological activity has not translated into consistent controlled clinical benefit and requires careful safety monitoring. Larger controlled studies using standardized clinical outcomes and reliable biomarkers are needed to define long-term effectiveness and optimize treatment selection.

## 1. Introduction

Niemann–Pick disease type C (NPC) is a rare neurodegenerative lysosomal disease with an incidence of one case per 150,000 live births [1]. The main characteristic feature is impaired intracellular lipid trafficking and subsequent accumulation of free cholesterol and glycosphingolipids in different organs such as the brain, liver or spleen [2]. Biallelic pathogenic variants in either NPC1 or NPC2 cause NPC, with NPC1 variants accounting for approximately 95% of affected families [2,3]. The symptoms of this autosomal recessive disease range from systemic to neurological and psychiatric deficits [4]. The most common symptom was hepatosplenomegaly, followed by developmental delay, neuroregression, ataxia, seizures, and vertical supranuclear gaze palsy [5]. Importantly, nearly one-third of patients presented with isolated visceral manifestations in the absence of neurological involvement. The heterogeneity of symptoms varies in terms of age at onset, severity and rate of progression [4]. NPC can be divided into early infantile (3 months to <2 years), late infantile (2 to 5 years), juvenile (6 to <15 years) and adolescent/adult-onset forms (≥15 years), which helps in the management of the disease, genetic counseling and prognosis [2,4]. The disease usually starts in early childhood and continues to get worse over time, which has a major impact on quality of life (QoL) [3]. But recently, there has been a rise in the cases reported for adolescent and adult-onset NPC [3]. The infantile form of NPC presents as an episode of prolonged neonatal cholestatic jaundice, which is an early sign of hepatosplenomegaly [1,3,4]. Delay in reaching the motor milestones is often another sign associated with the infantile form [1]. As the disease progresses, the neurological manifestations begin to dominate the systemic symptoms [1]. Characteristic neurological signs include vertical supranuclear gaze palsy (VSGP) and gelastic cataplexy [6]. Approximately 50% of patients with juvenile-onset NPC experience seizures, which can be refractory or poorly responsive to antiseizure medication. Other neurological manifestations include progressive dysmetria, cerebellar ataxia, hearing loss, dysarthria, dysphagia and cognitive impairment [1]. Seizures contribute substantially to morbidity and daily care requirements, making seizure control an important component of symptomatic management. The variable response to existing treatments highlights the need for improved antiseizure strategies and disease-modifying therapies targeting mechanisms that contribute to neuronal hyperexcitability [7]. It is noted that earlier onset of neurological symptoms is associated with more rapid disease progression and shorter life expectancy [3]. In a more advanced onset of the disease, psychiatric symptoms accompany the neurological symptoms [6,8]. Given the wide spectrum of symptoms, NPC is often diagnosed only after the onset of neurological symptoms, by which point ongoing neurodegeneration may have already caused irreversible damage [6].

In the past, treatment of NPC was mainly supportive, focusing primarily on symptomatic management [2]. The treatment of seizures in particular requires significant improvement. Drugs such as antiseizure, anticholinergic or antidepressants were used to alleviate the symptoms until some years ago, when several disease-modifying treatments emerged [6]. These treatments encompass substrate reduction therapies, pharmacological chaperones, drugs that target lysosomal function, and interventions targeting neuroinflammation and neuronal hyperexcitability [9].

Miglustat is an iminosugar substrate-reduction drug that was approved by the European Agency of Medicines in 2009 for the treatment of progressive neurological manifestations in pediatric and adult patients with NPC [4]. It slowed progression in a controlled trial and in registry data, with 70.5% of continuously treated patients improving or stabilizing across domains like ambulation, manipulation, and language [9]. Miglustat helps reduce the accumulation of glycosphingolipids in different organs such as the brain, which further helps in delaying the progression of the neurological manifestations in adult and pediatric patients [2,4]. This small iminosugar molecule reversibly inhibits the enzyme glucosylceramide synthase, which is an essential enzyme in glycosphingolipid synthesis [2,3]. In September 2024, arimoclomol was approved by the U.S. Food and Drug Administration (FDA), in combination with miglustat, for the treatment of neurological manifestations of NPC. Arimoclomol was initially characterized as a co-inducer of the heat shock response (HSR), enhancing the production of heat shock proteins (HSPs) under conditions of cellular stress and thereby supporting protein folding, cellular proteostasis, and lysosomal homeostasis [10]. It has also been suggested that arimoclomol may act by promoting transcription factor EB (TFEB) signaling, improving lysosomal function, and promoting lysosomal membrane stability [11]. Thus, this preserves cellular function and prevents cell death in cells experiencing lysosomal stress [10]. This was followed shortly thereafter by the development and approval of N-acetyl-L-leucine (NALL), which is an orally taken modified amino acid derivative. It exerts its therapeutic effects by modulating neuronal excitability and improving cerebellar function [8]. At the cellular level, NALL appears to help regulate intracellular pH, which often becomes unbalanced in NPC due to impaired lysosomal function [8,12]. It also seems to support mitochondrial energy metabolism by boosting adenosine triphosphate (ATP) production and reducing oxidative stress, particularly in Purkinje cells, which are critically affected in NPC [8]. NALL has emerged as a therapeutic option for its fast action on the symptomatic effects, notably on gait and coordination. Hydroxypropyl-β-cyclodextrin (HPβCD) can be administered intrathecally or intravenously and is intended to mobilize lysosomally sequestered cholesterol and restore intracellular cholesterol homeostasis. Adrabetadex, previously known as VTS-270, is administered intrathecally, while Trappsol Cyclo is administered intravenously [13]. It is a cyclic oligosaccharide that acts as a binding agent to cholesterol, aiding the clearance of accumulated lipids and hence restoring cholesterol homeostasis in peripheral organs. Adrabetadex in particular showed reduced progression and biomarker evidence of target engagement and decreased neuronal damage markers, as well as long-term stabilization in chronically treated patients [9]. Although HPβCD has not been formally approved, it has been widely studied and received orphan drug designation from the FDA in 2010 [6].

NPC is a debilitating disease that substantially affects the health span and QoL of the patient. Therefore, there is a significant unmet need to address the potential therapeutic options that can impact disease progression. However, we face a great challenge in the evaluation of therapeutic options for NPC given the limited number of patients and heterogeneity of NPC, which thus requires specific trial design considerations [13].

Given the expanding landscape of therapeutic approaches, this systematic review aims to evaluate and compare the efficacy and safety of different interventions that have been proven to show therapeutic effects in patients with NPC through a structured analysis of interventional clinical studies.

## 2. Methods

### 2.1. Eligibility Criteria

Studies taken into consideration for this review encompassed patients diagnosed with NPC of any age and sex. Eligible interventions included miglustat, arimoclomol, N-acetyl-L-leucine (NALL), hydroxypropyl-β-cyclodextrin (HPβCD), N-acetylcysteine (NAC), efavirenz, nizubaglustat, vorinostat, gene therapy, and any other pharmacological or biological strategy intended to treat NPC. A comparator was not mandatory because single-arm interventional studies were eligible. Where present, comparators included placebo, sham procedures, standard care, active treatment, historical controls, or participants’ pretreatment measurements. The outcomes contained within the study consisted of changes in neurological function assessed by scales such as the NPC clinical severity scale (NPCCSS), Scale for the Assessment and Rating of Ataxia (SARA) and Clinical Impression of Change in Severity (CI-CS). Other outcomes of interest included QoL, survival, biomarker changes, improvement in cognition and image-based endpoints. Eligible study designs comprised randomized controlled trials (RCTs), randomized crossover trials, and non-randomized interventional studies, including prospective open-label and uncontrolled before–after studies. ClinicalTrials.gov records were eligible only when results were publicly available and sufficient outcome data were reported. Animal studies, in vitro and in silico studies, observational studies without an investigator-assigned intervention, case reports, case series, reviews, study protocols, and registry records without posted results were excluded. Moreover, gray literature and review articles were ruled out. Only studies published in English were included.

### 2.2. Information Sources

A systematic literature search was conducted in PubMed and Scopus and the ClinicalTrials.gov trial registry from inception to 1 August 2026. The search strategy combined terms for NPC with general treatment concepts and intervention-specific terms. These included “therapeutic strategies,” “treatment,” “therapy,” “therapeutic intervention,” “management,” “clinical trial,” “pharmacological treatment,” and “disease-modifying therapy,” together with generic names, abbreviations, and brand or developmental names for miglustat, arimoclomol, HPβCD, NALL, NAC, efavirenz, nizubaglustat, and vorinostat.

### 2.3. Study Screening/Selection

Two reviewers independently screened and cross-checked through the aforementioned databases and registry. Screening was performed based on titles and abstracts against predetermined inclusion criteria, followed by full-text screening for the relevant studies. During the selection process, any disagreements were settled by discussion and, if required, by a third reviewer. We followed the Preferred Reporting Items for Systematic Reviews and Meta-Analyses (PRISMA) statement guidelines and performed all review procedures in accordance with the Cochrane Handbook for Systematic Reviews of Interventions. Figure 1 represents PRISMA flow diagram illustrating the identification, screening, eligibility assessment, and inclusion of studies in the systematic review. The review was designed according to the PICOS framework (Population, Intervention, Comparator, Outcomes, and Study Design) [14,15]. The protocol for this systematic review was registered in the International Prospective Register of Systematic Reviews (PROSPERO; Registration ID: CRD420250648780), available at: https://www.crd.york.ac.uk/prospero/display_record.php?ID=CRD420250648780, accessed on 1 August 2026.

### 2.4. Data Extraction

Two reviewers independently extracted data using a standardized extraction form developed in Microsoft Excel. For every study, the following data were gathered: name of the author, year of publication, country, study design and duration. Number of participants, age, gender, condition of interest and ethnicity were included for patient demographics. In interventional details, the treatment name, dose, dose regimen, adherence rate and dropout rates were mentioned. Furthermore, the comparator details encompassed the control interventions such as placebo, sham, standard care, active and historical control groups. Additionally, inclusion and exclusion criteria, outcome measures, assessment time points, numerical results, and adverse events were also extracted.

When publications or registry records reported overlapping participant cohorts, the relationship between the reports was documented and participants were counted only once when estimating the total number of participants represented across the review. However, linked reports were retained separately when they provided distinct interventions, follow-up periods, or outcome data. Any discrepancies in data extraction were discussed and resolved by the team. If needed, a third member stepped in to evaluate the discrepancy.

### 2.5. Risk of Bias

Two reviewers independently assessed the methodological quality of the included studies. Randomized trials were evaluated using the Cochrane Risk of Bias 2 (RoB 2) tool. The assessment covered five domains: bias arising from the randomization process, bias due to deviations from the intended interventions, bias due to missing outcome data, bias in measurement of the outcome, and bias in selection of the reported result. For crossover randomized trials, the additional domain concerning period and carryover effects was assessed. Each domain and the overall risk-of-bias judgment were classified as “Low risk,” “Some concerns,” or “High risk.”

Uncontrolled before–after studies were evaluated using the National Heart, Lung, and Blood Institute (NHLBI) Quality Assessment Tool for Before–After (Pre–Post) Studies With No Control Group. The tool contains 12 items addressing the clarity of the research objective, participant selection, sample-size justification, intervention delivery, outcome measurement, assessor blinding, completeness of follow-up, and appropriateness of the statistical analysis. Item-level responses were recorded as “Yes,” “No,” “Cannot determine,” “Not reported,” or “Not applicable,” as appropriate. An overall methodological quality rating of “Good,” “Fair,” or “Poor” was assigned through qualitative appraisal rather than by calculating a numerical score. Disagreements between the two reviewers were resolved through discussion and consensus.

### 2.6. Data Synthesis

As substantial clinical and methodological heterogeneity was identified across interventions, study designs, outcome measures, assessment time points, and follow-up durations, quantitative meta-analysis was not considered appropriate. Findings were therefore synthesized narratively and grouped according to the intervention evaluated. The synthesis considered the direction and magnitude of treatment effects, consistency across studies, study design, methodological quality, duration of follow-up, and adverse-event profile.

**Table 1:** Studies retrieved from the databases for miglustat.

S.NO	STUDY TITLE & DESIGN	INTERVENTIONS & COMPARATOR	POPULATION DEMOGRAPHICS	PRIMARY OUTCOMES	RESULTS	ADVERSE EVENTS (AE)
1.	Miglustat for treatment of Niemann–Pick C disease: a randomized controlled study [16] **Study design:** 12-month randomized, open-label, controlled trial in participants aged ≥12 years, with a parallel non-randomized pediatric cohort	**Intervention**: Miglustat (n = 32) **Comparator**: Standard Supportive Care (n = 9)	**Age**: Miglustat group—12–42 years; Control group—13–32 years; Pediatric group—4–11 years **Sex**: Miglustat group—9 male/11 female Control group—, 5 male/4 female; Pediatric group—5 male/7 female	Change from baseline in Horizontal Saccadic Eye Movement asymptotic peak velocity (HSEM-α)	Adjusted HSEM-α difference was −0.518 ms/degree (95% CI −1.125 to 0.089; *p* = 0.091). Excluding six benzodiazepine users, the difference was −0.718 (95% CI −1.349 to −0.088; *p* = 0.028). Pediatric mean change was −0.465. Cookie swallowing favored miglustat (*p* = 0.044); ambulation difference was −0.715 (*p* = 0.052); MMSE difference was not significant (*p* = 0.165).	Common in miglustat group: diarrhea (85%), flatulence (70%), weight loss (65%), abdominal pain (50%); less frequent in controls
2.	Long-Term Miglustat Therapy in Children with Niemann–Pick Disease Type C [3] **Study design:** Non-controlled, Open-Label Extension	**Intervention**: Miglustat (n = 12) **Comparator:** None	**Age**: 4–11 years **Sex**: 5 male/7 female	Change in HSEM-α from baseline to months 12 and 24.	Mean HSEM-α was 2.181 ms/degree at baseline, 1.692 at Month 12, and 2.106 at Month 24 (n = 9). Overall disease stability occurred in 8/10; HSEM-α improved or remained stable in 6/9, ambulation in 8/10, and cookie swallowing in 9/10. No inferential statistical testing was performed.	Diarrhea (67%), tremor (58%), headache (50%), vomiting, fatigue; most events mild/moderate
3.	Miglustat in adult and juvenile patients with Niemann–Pick disease type C: Long-term data from a clinical trial [2] **Study design:** Non-controlled, Open-Label Extension	**Intervention**: Miglustat (n = 21 for Extension; n = 15 for continued Extension) **Comparator:** None	**Age**: 12–42 years **Sex**: 14 male/15 female	HSEM-α at months 12 and 24.	HSEM-α improved initially but remained broadly stable at Month 24. Overall disease stability occurred in 13/19 (68.4%); HSEM-α improved or remained stable in 11/18, swallowing in 15/19, ambulation in 17/19, and cognition in 14/18. No statistical significance testing was undertaken.	Diarrhea (89%), weight loss (75%), flatulence (64%), tremor (57%)
4.	Miglustat therapy in the French cohort of pediatric patients with Niemann–Pick disease type C [4] **Study design:** Prospective, open-label cohort study	**Intervention**: Miglustat (n = 20) **Comparator:** None	**Age**: 2 months–14.8 years **Sex**: 11 female/9 male	Change in the four-domain NP-C disability scale assessing ambulation, manipulation, language, and swallowing	Neurological improvement or stabilization occurred in 1/8 early-infantile, 6/8 late-infantile, and 1/3 juvenile-onset patients, indicating greater benefit in late-infantile and juvenile disease.	Miglustat-related AEs occurred in 15/20 (75%), mainly early diarrhea, abdominal pain, anorexia, and weight loss. Three discontinued because of persistent AEs and limited benefit. Three disease-related deaths occurred.
5.	Long-term follow-up to evaluate the efficacy of miglustat treatment in Italian patients with Niemann–Pick disease type C [1] **Study design**: Partly single-arm, open-label clinical trial and partly observational study	**Intervention**: Miglustat (n = 25) **Comparator:** None	**Age**: 7 months–44 years **Sex**: 9 male/16 female	Improvement or stabilization of neurological manifestations and swallowing, assessed using the Mean Composite Severity Score (MCSS)	At 24 months, improvement or stabilization occurred in 86% for gait, 80% for dystonia, 69% for dysarthria, 61% for dysmetria, and 68.5% for cognition. Swallowing improved or remained stable in 55–65%. At 48–96 months, benefits persisted in approximately 41–55% for neurological outcomes. Early treatment was associated with greater MCSS stability: 4/7 versus 1/16 treated later.	Common AEs were diarrhea and weight loss; less frequent events included tremor, behavioral problems, thrombocytopenia, and leukopenia. One serious epileptic seizure was considered unrelated to treatment.

**Table 2:** Studies retrieved from the databases for arimoclomol.

S.NO	STUDY TITLE & DESIGN	INTERVENTIONS & COMPARATOR	POPULATION DEMOGRAPHICS	PRIMARY OUTCOMES	RESULTS	ADVERSE EVENTS (AE)
1.	Efficacy and safety of arimoclomol in Niemann–Pick disease type C: Results from a double-blind, randomized, placebo-controlled, multinational phase 2/3 trial of a novel treatment [10] **Study design:** Prospective, randomized, double-blind, placebo-controlled, multinational Phase II/III trial; NCT02612129	**Intervention**: Arimoclomol (n = 34) **Comparator**: Placebo (n = 16)	**Age**: 2–18 years **Sex**: 26 female/24 male	Change in total NPCCSS score from baseline to 12 months	Mean five-domain NPCCSS change was 0.76 with arimoclomol versus 2.15 with placebo; difference −1.40 (95% CI −2.76 to −0.03; *p* = 0.046), representing 65% less progression. In the concomitant-miglustat subgroup, the difference was −2.06 (95% CI −3.49 to −0.63; *p* = 0.006). Responder and CGI-I differences were not significant.	AEs: 88.2% (30/34) arimoclomol vs. 75.0% (12/16) placebo; SAEs: 14.7% (5/34) vs. 31.3% (5/16); 2 TSAEs (urticaria, angioedema)
2.	Long-term efficacy and safety of arimoclomol in Niemann–Pick disease type C: Final results of the phase 2/3 NPC-002 48-month open-label extension trial [17] **Study design:** Single-arm, 48-month open-label extension (OLE)	**Intervention**: Arimoclomol (n = 41) **Comparator**: None	**Age**: 2–18 years **Sex**: 21 female/20 male	Change from OLE baseline in the five-domain NPCCSS and rescored four-domain NPCCSS (R4DNPCCSS) through Month 48	Among 29 completers, mean 48-month change was 3.2 ± 4.8 for the five-domain NPCCSS and 2.7 ± 4.2 for the R4DNPCCSS, corresponding to annual progression of approximately 0.8 and 0.7 points. In placebo-to-arimoclomol participants, annual progression decreased from 2.0 to 0.1 and from 1.9 to 0.2, respectively. The uncontrolled design limits causal interpretation.	AEs occurred in 92.7% and serious AEs in 36.6%. Twenty of 460 AEs were treatment-related; one was serious moderate proteinuria. Four AEs caused discontinuation. Two deaths were unrelated to treatment.
3.	Arimoclomol in infants with Niemann–Pick disease type C: Results from the phase 2/3 open-label pediatric substudy [18] **Study design:** Phase 2/3, multicenter, open-label pediatric substudy of NPC-002	**Intervention**: Arimoclomol (n = 5) **Comparator**: None	**Age**: 14–23 months **Sex**: 3 female/2 male	Safety and tolerability assessed through AEs, laboratory tests, and vital signs	Five infants enrolled. Four remained for >12 months; two completed and three withdrew. Development improved in one, remained broadly stable in two, declined in one, and was unavailable after baseline in one. Pharmacokinetic exposure was comparable with older pediatric patients; biomarker changes were variable.	All five participants experienced AEs. Fifteen serious AEs occurred in two participants, none treatment-related. Elevated alanine aminotransferase (ALT) and aspartate aminotransferase (AST) in one participant were probably treatment-related and resolved after interruption. No deaths occurred.

**Table 3:** Studies retrieved from the databases for NALL.

S.NO	STUDY TITLE & DESIGN	INTERVENTIONS & COMPARATOR	POPULATION DEMOGRAPHICS	PRIMARY OUTCOMES	RESULTS	ADVERSE EVENTS (AE)
1.	Efficacy and safety of N-acetyl-L-leucine in Niemann–Pick disease type C [8] **Study design:** Phase II, multinational, open-label, rater-blinded clinical trial	**Intervention**: NALL (n = 33) **Comparator**: None	**Age**: 7–64 years **Sex**: 24 male/9 female	CI-CS	Primary modified intention-to-treat (mITT) analysis included 32 patients. CI-CS difference was 0.86 ± 2.52 (90% CI 0.25–1.75; *p* = 0.029), reflecting improvement during treatment and deterioration during washout. SARA changed by −1.19 during treatment (*p* = 0.001) and +1.45 during washout (*p* = 0.002).	Seven treatment-related AEs occurred in four participants and were transient. Two participants withdrew after unrelated events. No treatment-related serious AEs or deaths occurred.
2.	Trial of N-Acetyl-l-Leucine in Niemann–Pick Disease Type C [12] **Study design**: Phase III, randomized, double-blind, placebo-controlled, crossover trial	**Intervention**: NALL (n = 30) **Comparator**: Placebo (n = 30)	**Age**: 5–67 years **Sex**: 33 male/27 female	Change in total SARA score after 12 weeks of NALL vs. placebo (crossover design)	SARA changed by −1.97 ± 2.43 with NALL versus −0.60 ± 2.39 with placebo; least-squares difference −1.28 (95% CI −1.91 to −0.65; *p* < 0.001). Modified SARA difference was −0.96; mDRS difference −0.029. Investigator and caregiver CGI-I favored NALL; SCAFI and exploratory NPC-CSS differences were smaller.	NALL: 79 AEs in 36/60 participants; placebo: 75 AEs in 30/59. Three transient NALL-related AEs occurred. One unrelated withdrawal and death from aspiration pneumonia followed a planned gastrostomy procedure.
3.	Disease-Modifying, Neuroprotective Effect of N-Acetyl- l -Leucine in Adult and Pediatric Patients with Niemann–Pick Disease Type C [19] **Study design**: Open-label, ongoing, multinational, multicenter extension phase of the randomized, double-blind, placebo-controlled crossover	**Intervention**: NALL (n = 53) **Comparator**: Natural-history cohort (n = 32)	**Age**: 5–67 years **Sex**: Female 24/male 29	Change from baseline on modified 5-domain NPC-CSS at 12 and 18 months vs. historical cohort	Five-domain NPC-CSS difference versus expected natural history was −1.77 at Month 12 (95% CI −3.05 to −0.48; *p* = 0.009) and −2.20 at Month 18 (95% CI −4.06 to −0.35; *p* = 0.023). Fifteen-domain differences were −1.87 and −2.37, respectively. SARA improved by −1.88 at Month 12 and −1.64 at Month 18 (both *p* < 0.001 versus no change).	No TEAEs led to discontinuation; no TEAEs in >10% of patients; none assessed as related to NALL; no drug-related SAEs or deaths; labs, vitals, and ECG normal or nonsignificant

**Table 4:** Studies retrieved from the databases for HPβCD.

S.NO	STUDY TITLE & DESIGN	INTERVENTIONS & COMPARATOR	POPULATION DEMOGRAPHICS	PRIMARY OUTCOMES	RESULTS	ADVERSE EVENTS (AE)
1.	Intrathecal 2-hydroxypropyl-β-cyclodextrin decreases neurological disease progression in Niemann–Pick Disease, type C1: an ad hoc analysis of a non-randomized, open-label, phase 1/2 trial [13] **Study design**: Open-label, Phase 1/2a trial	**Intervention**: Intrathecal HPβCD (n = 17) **Comparator**: Historical control group from the NIH Natural History study (n = 21)	**Age**: 4–24 years **Sex**: 16 male/19 female	Safety and tolerability of HPβCD was the primary objective, and 24(S)- hydroxycholesterol (24(S)-HC) response was the primary pharmacodynamic (PD) objective.	Plasma 24(S)-hydroxycholesterol response was greater with HPβCD at 900 mg (*p* = 0.0063) and 1200 mg (*p* = 0.0037). Cerebrospinal fluid (CSF) fatty acid-binding protein 3 and calbindin D decreased. In the NIH cohort, total NSS progression was 1.22 versus 2.92 points/year in historical controls (*p* = 0.0002); NSS excluding hearing progressed by 0.69 versus 2.67 points/year (*p* < 0.0001). Seven of 14 treated participants versus 0/21 controls were stable or improved (*p* = 0.0005).	Expected: Ototoxicity- Mid-to-high-frequency hearing loss (100%), post-lumbar puncture headache (64%) Unexpected: Transient unsteadiness/fatigue post-dosing >600 mg
2.	Long-Term Neuropsychological Outcomes from an Open-Label Phase I/IIa Trial of 2-Hydroxypropyl-β-Cyclodextrins (VTS-270) in Niemann–Pick Disease, Type C1 [20] **Study design**: Open-label, dose-escalation, Phase 1/2a study	**Intervention**: Intrathecal HPβCD (n = 14) **Comparator**: None	**Age**: 4 to 23 years **Sex**: 7 female/7 male	Full Scale IQ (FSIQ); Verbal IQ; Nonverbal IQ; Adaptive behavior (Vineland-II composite and domain scores)	FSIQ declined by −1.28 points/year (*p* = 0.076). Vineland-II composite standard score declined by −1.76 points/year (*p* = 0.011); socialization standard score also declined (*p* = 0.04). Positive but nonsignificant domain age-equivalent slopes suggested continued skill acquisition at a slower-than-expected rate. Memory data were insufficient for parametric analysis.	Mid- to high-frequency hearing loss was observed in all participants
3.	Intravenous 2-hydroxypropyl-β-cyclodextrin (Trappsol^®^ Cyclo™, Cyclo Therapeutics, Inc., Gainesville, FL, USA ) demonstrates biological activity and impacts cholesterol metabolism in the central nervous system and peripheral tissues in adult subjects with Niemann–Pick Disease Type C1: Results of a phase 1 trial [6] **Study design:** Phase 1, randomized, double-blind, parallel-group study	**Intervention**: HPβCD 1500 mg/kg (n = 6) **Comparator**: HPβCD 2500 mg/kg (n = 7)	**Age**: 18 to 69 years **Sex**: 5 female/8 male	Safety and tolerability, pharmacokinetics (PK) in plasma	Ten of 13 completed treatment. Plasma half-life was approximately 2 h; maximum concentration was higher with 2500 mg/kg. Peripheral biomarkers and liver filipin staining improved. Cerebrospinal fluid total tau decreased in 8/10. The 17-domain NPC-CSS remained broadly stable in the 1500 mg/kg group but increased in the high-dose group; no formal efficacy testing was conducted.	Forty treatment emergent AEs occurred. Hearing loss occurred in 6/13: 1/6 at 1500 mg/kg and 5/7 at 2500 mg/kg. Eight serious AEs occurred in four high-dose participants. Three participants withdrew.
4.	Long-term administration of intravenous Trappsol^®^ Cyclo™ (HP-β-CD) results in clinical benefits and stabilization or slowing of disease progression in patients with Niemann–Pick disease type C1: Results of an international 48-week Phase I/II trial [21] **Study design**: Multicentre, Phase I/II, randomized, double-blind, parallel-group, NCT02912793	**Intervention**: Intravenous HPβCD (n = 12) **Comparator**: None	**Age**: 2 to 39 years **Sex**: 5 female/7 male	Proportion of participants achieving a ≥1-point improvement in at least two 17D-NPC-CSS domains at Week 48.	Nine of 12 participants completed 48 weeks. The primary responder criterion was met by 8/9 completers (88.9%); 6/8 improved in ≥1 quality-of-life-relevant domain. Clinicians rated 7/9 as improved and 2/9 as stable; all nine were improved or stable by PGIC. Mean SARA scores improved in seven of eight domains. Liver size improved in 2/6 and spleen size improved or normalized in 2/9 evaluable participants, with no worsening of organomegaly. HPβCD was detected in CSF. In limited biomarker datasets, 24(S)-HC peaked 22% above baseline after the first infusion and N-palmitoyl-O-phosphocholineserine (PPCS) decreased by 61.3% at Week 16. Analyses were descriptive, with no formal inferential efficacy testing.	All 12 participants experienced ≥1 TEAE (185 events); related TEAEs occurred in 7/12, grade ≥3 in 5/12, and serious TEAEs in 5/12. No deaths or treatment-related withdrawals occurred. Common TEAEs included seizures, rash, rhinitis, and cataplexy, mostly unrelated to treatment. Hearing loss occurred in 2 participants, with transient changes in 3.

**Table 5:** Studies retrieved from the databases for efavirenz.

S.NO	STUDY TITLE & DESIGN	INTERVENTIONS & COMPARATOR	POPULATION DEMOGRAPHICS	PRIMARY OUTCOMES	RESULTS	ADVERSE EVENTS (AE)
1.	Efficacy and safety of efavirenz in Niemann–Pick disease type C [22] **Study design:** Phase II, single-center, single-arm, open-label trial	**Intervention:** Efavirenz (n = 16) **Comparator**: None	**Age**: 15–60 years **Sex**: 11 males/5 females	Proportion of responders at Week 52, defined as no deterioration in ≥2 of 3 cognitive domains: CDR-SB, Free and Cued Selective Reminding Test (FCSRT), and executive-function tests	All 16 participants responded at Week 52 (100%; 95% CI 79.4–100.0%); 15/16 responded earlier. This represented non-deterioration in ≥2 domains, not universal improvement. Post hoc favorable-response and unfavorable-response groups differed mainly by disease duration. SARA and PDS were broadly stable; EAT-10 and WAIS-III improved. Cerebrospinal fluid amyloid-beta increased and tau remained stable.	Three AEs occurred in one participant: traumatic brain injury, splenic laceration following an ataxia-related fall, and myelocytic leukemia. All were considered unrelated to efavirenz; no treatment-related serious AEs occurred.

**Table 6:** Studies retrieved from the databases for nizubaglustat.

S.NO.	STUDY TITLE & DESIGN	INTERVENTIONS & COMPARATOR	POPULATION DEMOGRAPHICS	PRIMARY OUTCOMES	RESULTS	ADVERSE EVENTS (AE)
1.	The RAINBOW study: a phase 2 trial evaluating the 3-month pharmacokinetic and pharmacodynamic data and 18-month clinical and safety outcomes of nizubaglustat in GM2 gangliosidoses or Niemann–Pick type C disease [23] **Study design:** Phase II, multicenter trial with a 12-week randomized, double-blind, placebo-controlled phase and optional active-treatment extension	**Intervention:** Nizubaglustat 3 mg/day (n = 5) or 9 mg/day (n = 4) **Comparator:** Placebo (n = 4)	**Age**: 12–38 years **Sex**: 7 male/6 female	Pharmacokinetics (PK); pharmacodynamics (PD) of C16/C18 ceramides and glucosylceramides; 12-week safety	Absorption peaked at 2–6 h. The 9 mg dose produced higher exposure and reduced C16 and C18 glucosylceramides by up to 87% and 47%, respectively; ceramides were unchanged. At 15–18 months, 4/11 improved, 1 remained stable, and 6 worsened on SARA; 6/11 improved on either SARA or INAS.	At Week 12, 10/13 experienced only mild or moderate treatment-emergent AEs. 44 long-term events, skin events occurred in 7/13. One unrelated serious liver abscess occurred. No deaths or AE-related discontinuations occurred.

**Table 7:** Studies retrieved from the ClinicalTrials.gov.

S.NO	STUDY TITLE	INTERVENTIONS & COMPARATOR	POPULATION DEMOGRAPHICS	PRIMARY OUTCOMES	RESULTS	ADVERSE EVENTS
1.	A Phase 2b/3 Prospective, Randomized, Double-Blind, Sham-Controlled 3-Part Trial of VTS-270 (2-hydroxypropyl-β-cyclodextrin) in Subjects With Neurologic Manifestations of Niemann–Pick Type C1 (NPC1) Disease [24] **Study design**: Randomized, double-blind, sham-controlled Parts A/B; NCT02534844	**Intervention:** HP*β*CD (Adrabetadex/VTS-270) (n = 38) **Comparator**: Sham control (n = 18)	**Age**: 4–21 years **Sex**: 30 male/26 female	Change in 4-Item Composite NPC-SS Score (Week 52)	Four-item NPC-SS change was −0.1 ± 1.55 with sham versus 0.0 ± 2.45 with adrabetadex. CGIC responders were 7/18 versus 23/38; NPC-SS responders were 13/18 versus 22/38. Median time to worsening was 118 versus 169 days. No comparative *p*-values were posted.	Treatment-emergent AEs occurred in 17/18 (94.4%) versus 37/38 (97.4%); serious AEs in 3/18 (16.7%) versus 19/38 (50.0%). One death occurred in each group.
2.	A Phase 2b/3 Prospective, Randomized, Double-blind, Sham-controlled Trial of VTS-270 (2-hydroxypropyl-β-cyclodextrin) in Subjects with Neurologic Manifestations of Niemann–Pick Type C1 (NPC1) Disease [25] **Study design:** Open-label Part C continuation; NCT04958642	**Intervention:** HP*β*CD (Adrabetadex) (n = 66) **Comparator**: None	**Age**: 4–21 years **Sex**: 34 male/32 female	Participants experiencing treatment-emergent AEs or serious AEs over ≤5 years	Treatment-emergent AEs occurred in 18/18 treatment-naïve, 13/13 Phase I, and 34/35 Parts A/B participants: overall 65/66 (98.5%). Serious AEs occurred in 9/18, 7/13, and 20/35, respectively: overall 36/66 (54.5%).	Two deaths occurred: one treatment-naïve participant and one Parts A/B continuation participant. Reported serious events included infections, respiratory failure, seizures, procedural complications, hemorrhage, and hearing disorders.
3.	A Phase 2b/3 Open-label Trial of VTS-270 (2-hydroxypropyl-β-cyclodextrin) in Subjects With Neurologic Manifestations of Niemann–Pick Type C1 Disease Previously Treated Under Protocol VTS301 [26] **Study design:** Open-label continuation; NCT03879655	**Intervention:** HP*β*CD (Adrabetadex) (n = 2) **Comparator**: None	**Age**: <18 years **Sex**: Not reported	Participants experiencing treatment-emergent AEs or serious AEs through Week 156	Both participants experienced treatment-emergent AEs; neither experienced a serious AE.	No deaths; no SAEs reported
4.	Open-label Evaluation of Adrabetadex in Patients with Neurologic Manifestations of Niemann–Pick Type C Disease (NPC) [27] Study design: Open-label, Interventional; NCT03643562	**Intervention:** HP*β*CD (Adrabetadex) (n = 27) **Comparator**: None	**Age**: 16–21 years **Sex**: 16 male/11 female	Participants experiencing treatment-emergent AEs or serious AEs through Week 134	Treatment-emergent AEs occurred in 9/9 treatment-naïve and 17/18 previously treated participants: overall 26/27 (96.3%). Serious AEs occurred in 5/9 and 9/18, respectively: overall 14/27 (51.9%).	Ventricular extrasystoles, Deafness, Constipation
5.	Phase 1/2 Study of Vorinostat Therapy in Niemann–Pick Disease, Type C1 [28] Study design: Phase I/II, open-label, single-group trial; NCT02124083	**Intervention:** Vorinostat (n = 12) **Comparator**: None	**Age**: 18–65 years; **Sex**: 6 male/6 female	Completion of the 200 mg and 400 mg treatment phases at three months	All 12 completed the 200 mg phase and 11/12 completed the 400 mg phase. Cathepsin D was 648.2 ± 266.0 ng/mL at baseline and 625.3 ± 183.8 at six months. Galectin-3 was 6.667 ± 2.303 and 6.565 ± 4.775 ng/mL, respectively.	Serious AEs occurred in 5/12 (41.7%); non-serious AEs in 11/12 (91.7%). Serious events included vomiting, Epstein–Barr viremia, fever, cerebrospinal fluid leakage, abnormal liver tests, and aspiration pneumonia. No deaths occurred.
6.	Biomarker Validation for Niemann–Pick Disease, Type C: Safety and Efficacy of N-Acetyl Cysteine [29] Study design: Randomized, triple-blind, placebo-controlled crossover trial; NCT00975689	**Intervention:** NAC (n = 17) **Comparator**: Placebo (n = 18)	**Age**: 12 months–65 years **Sex**: 17 female/18 male	Oxysterol levels at six months	Mean oxysterol concentration was 28.09 ± 2.15 ng/mL during NAC and 27.64 ± 2.04 ng/mL during placebo.	Thirty of 35 completed the crossover. Serious AEs occurred in 3/17 participants assigned placebo→NAC and 5/18 assigned NAC→placebo; events were reported by sequence rather than treatment period.
7.	Evaluation of the safety and efficacy of miglustat for the treatment of Chinese patients with Niemann–Pick disease type C: A prospective, open-label, single-arm, phase IV trial [30] Study design: Prospective, multicenter, open-label, single-arm Phase IV trial; NCT03910621	**Intervention:** Miglustat (n = 17) **Comparator**: None	**Age**: 6–33 years **Sex**: 9 male/8 female	HSEM saccadic peak acceleration (Week 52)	At Week 52 (n = 13), peak acceleration increased 19.2% (95% CI 11.7–26.7), mean velocity increased 12.5%, peak duration decreased 6.5%, and regression slope decreased 15.6%. Pineda scores generally improved numerically or remained stable.	All 17 experienced treatment-emergent AEs; 12/17 had treatment-related AEs. Diarrhea occurred in 12/17, upper respiratory infection in 7/17, hyperuricemia in 4/17, and epistaxis in 3/17. Five experienced serious AEs. Two unrelated fatal AEs caused discontinuation.

## 3. Results

### 3.1. Study Selection

Using our search criteria, 12,298 records were identified through the initial database and registry searches (PubMed: 6087; Scopus: 5876; ClinicalTrials.gov: 335). After removing 9139 duplicates, 3159 records underwent title screening, of which 2297 were excluded. The abstracts of the remaining 862 records were screened, and 815 were excluded. Full texts were sought for 47 reports; one could not be retrieved, leaving 46 reports for eligibility assessment. Twenty-two reports were excluded because they had no available results (n = 10), were case series (n = 6), observational studies (n = 2), study protocols (n = 2), or case reports (n = 2). Consequently, 24 studies were included in the systematic review (Figure 1). After accounting for known overlap among parent trials, extension studies, rollover cohorts, historical controls, and linked registry records, the included evidence represented approximately 475 unique participants.

### 3.2. Study Characteristics

Six trials investigated miglustat, three focused on arimoclomol, three evaluated NALL, and eight assessed various formulations and routes of HPβCD. One study each evaluated efavirenz, nizubaglustat, vorinostat, and N-acetylcysteine (NAC). The evidence base was therefore concentrated primarily on HPβCD and miglustat, whereas the other interventions were supported by fewer studies. The study designs included randomized parallel-group and crossover trials, randomized active-dose-ranging studies, prospective open-label single-arm trials, dose-escalation studies, and open-label extension or rollover studies. Participants’ ages were variable, ranging from 2 months to 69 years of age, with study sizes varying from 2 [26] to 66 [25] participants. There were certain studies that focused only on the pediatric cohort, while others included broad age ranges.

Planned active-treatment periods ranged from 6 weeks to 96 months. The primary outcomes differed substantially among studies and included horizontal saccadic eye movement (HSEM) parameters, different versions of the NPC Clinical Severity Scale (NPCCSS), the Scale for the Assessment and Rating of Ataxia (SARA), the Clinical Impression of Change in Severity (CI-CS), pharmacokinetic and biomarker outcomes, and safety or tolerability endpoints. Secondary outcomes included swallowing, ambulation, cognition, neurological function, quality of life (QoL), organ volumes, biochemical markers, and adverse events (AEs), including gastrointestinal symptoms and hearing impairment. Full study-level characteristics and results are presented in Table 1, Table 2, Table 3, Table 4, Table 5 and Table 6 for the published studies and Table 7 for the ClinicalTrials.gov records. The ClinicalTrials.gov records in Table 7 represent publicly available registry results for which no corresponding peer-reviewed publication was identified at the time of the updated search. A meta-analysis was not performed because of substantial clinical and methodological heterogeneity in the interventions, study designs, populations, outcome definitions, measurement scales, and follow-up periods. The findings were therefore synthesized narratively by intervention and interpreted in the context of study design and methodological quality.

### 3.3. Risk of Bias Assessment

Risk of bias and methodological quality were assessed using tools appropriate to each study design. RoB 2 was applied to seven randomized results, comprising five parallel-group results and two crossover results. The NIH/NHLBI Quality Assessment Tool for Before–After (Pre–Post) Studies with No Control Group was applied to 17 uncontrolled studies. Figure 2 represents the risk of bias assessment of the included studies.

Among the five parallel-group randomized results, three were judged to have a low risk of bias across all domains, while two were judged to have some concerns. These concerns arose primarily from insufficient information about the randomization process and uncertainty concerning the selection of the reported result. Both randomized crossover results were judged to have some concerns. In one crossover study, concerns related to the selection of the reported result, whereas the other had additional concerns regarding randomization, missing outcome data, and possible period or carryover effects. None of the randomized results were classified as having a high risk of bias.

Of the 17 uncontrolled studies assessed using the NIH/NHLBI tool, one was rated as having good methodological quality, nine were rated as fair, and seven were rated as poor. The Bremova-Ertl et al. [8] study received the good-quality rating because of its prospective design, high participant retention, standardized outcome assessment, blinded central raters, and clearly described intervention. Studies rated as fair generally had clearly stated objectives, adequately described interventions, and standardized outcome measures but commonly lacked sample-size justification, blinded outcome assessment, repeated pre-intervention measurements, or sufficiently complete follow-up. Poor-quality ratings were primarily associated with small or highly selected samples, substantial or incompletely reported attrition, inconsistent treatment exposure, lack of blinded assessment, and inadequate information regarding intervention delivery or statistical analysis.

Overall, the randomized evidence demonstrated more favorable internal-validity assessments than the uncontrolled before–after evidence. Although the uncontrolled studies provided valuable information regarding safety, long-term treatment, and outcomes in rare patient populations, their findings should be interpreted cautiously because the absence of concurrent comparison groups limits the extent to which observed changes can be attributed to the interventions. These methodological limitations were considered when interpreting the strength and consistency of the review findings.

### 3.4. Therapeutic Outcomes by Interventions

#### 3.4.1. Miglustat

Six studies evaluated miglustat, including one randomized, open-label comparison with standard supportive care and five uncontrolled or extension studies [1,2,3,4,16,30,31]. Adults and adolescents generally received 200 mg orally three times daily, while pediatric doses were adjusted according to body surface area [1,2,3,4,16]. The pediatric and adult extension publications included participants from the original randomized study and therefore provided longer follow-up of linked cohorts rather than independent participant populations. 

The study conducted by Patterson et al. assessed the efficacy and safety of the drug across two age cohorts consisting of adolescents/adults aged ≥12 years and children aged 4–11 years. The participants aged ≥12 years were randomized to receive either miglustat (n = 20) or standard supportive care (n = 9), while a parallel pediatric cohort received open-label miglustat from baseline (n = 12). Standard care consisted of pharmacotherapy, physical, speech, and occupational therapy, which were tailored to each patient’s clinical requirement, without administration of miglustat or any other disease-modifying agents. After 12 months, miglustat slowed the decline in the primary outcome, which was the horizontal saccadic eye movement (HSEM-α), compared to standard care (−0.431 vs. +0.074 ms/deg; Δ = −0.505). The difference reached statistical significance after excluding patients on benzodiazepines, which are well-known to affect eye movements (*p* = 0.028). Among the secondary outcomes, swallowing of cookies favored miglustat (*p* = 0.044), while differences in HSEM-β (*p* = 0.834), ambulation (*p* = 0.052), and Mini-Mental State Examination (MMSE) scores (*p* = 0.165) were not statistically significant. Auditory acuity remained broadly stable in the miglustat group but declined in the standard-care group. The most common AEs noted were diarrhea (85%), flatulence (70%), weight loss (65%), and abdominal pain (50%), which generally decreased over time. Four serious adverse events (SAEs) occurred among miglustat-treated participants, but none was considered treatment-related. No deaths occurred during the study, but two patients aged ≥12 years and one patient aged 4–11 years discontinued the treatment due to AEs [16].

To further evaluate the long-term effects of the drug, an open-label extension for the same pediatric cohort was conducted by Patterson et al. This study followed the pediatric cohort for up to 24 months, with safety monitoring extending up to 52 months. By the end of 24 months, 8/10 (80%) of the participants had shown stable disease progression based on specific neurological criteria. Initial signs of improvement were seen in HSEM-α, with the mean value improving from 2.181 to 1.692 ms/deg at 12 months but returning close to baseline by 24 months (2.106 ms/deg). Mean HSEM-β increased from 28.96 ms/degree at baseline to 33.66 ms/degree at 12 months and remained similar at 24 months (33.47 ms/degree), indicating an early change followed by little further deterioration. Ambulation, nevertheless, worsened gradually, with the mean Standard Ambulation Index increasing from 2.0 at baseline to 2.3 at 12 months and 2.6 at 24 months. Beyond eye movements, swallowing showed consistently preserved function, as 80% of the patients remained stable across the study. Cognitive function, measured using MMSE, and auditory acuity were assessed at multiple time points, but no statistically significant changes were reported. The safety profile was similar, with the presence of diarrhea (67%) as an AE, along with tremor (58%) and headache (50%), followed by vomiting and fatigue [3].

The pivotal RCT and subsequent open-label extension by Patterson et al. [3,16] suggested that miglustat may slow or stabilize neurological decline in NPC. Hence, building on these encouraging findings, several open-label studies were launched across Europe to further assess miglustat’s use in controlled trial environments and explore its long-term benefits. One such open-label extension study, by Wraith et al. [2], reported the corresponding extension in the juvenile and adult cohort aged ≥12 years. Participants who completed the 12-month randomized phase were eligible to continue treatment, regardless of their initial group. Those previously receiving standard care began miglustat during the extension. A total of 21 patients completed 12 months of therapy, and 15 continued for 24 months. Safety data were gathered for up to 66 months. HSEM-α improved by 12 months (from 3.06 to 2.87) and remained stable at 24 months (3.27), indicating preserved ocular motor function. HSEM-β values increased slightly, suggesting a mild early decline, but leveled off, indicating stabilization over time. Swallowing ability remained stable or improved in 86% of patients at 12 months and in 93% by 24 months, supporting earlier findings of miglustat’s effect on bulbar function. Ambulation measured using the Standard Ambulation Index remained largely unchanged over 24 months (mean score: 2.13 to 2.40), and MMSE scores showed minimal variation (22.94 at baseline to 24.06 at 12 months). Importantly, 13 out of 19 patients (68%) who received at least 12 months of therapy were categorized as having stable disease, defined as no worsening in ambulation, swallowing, or cognition, or only isolated deterioration in HSEM-α. The safety profile remained consistent throughout. Among the 28 participants who received at least one dose of miglustat, the most frequently reported treatment-emergent adverse events during safety follow-up of up to 66 months were diarrhea (89.3%), weight decrease (75.0%), flatulence (64.3%), and tremor (57.1%). A few patients discontinued treatment due to side effects or personal reasons, and one was withdrawn for non-compliance. There were no miglustat-related deaths that had occurred [2].

In parallel, Héron et al. [4] undertook a prospective, multicentre, open-label study in France that enrolled 20 pediatric patients with genetically confirmed NPC, including 19 with NPC1 mutations and 1 with an NPC2 mutation. Participants were followed for a median of approximately 1–2 years and were grouped by neurological-onset phenotype. Stabilization or improvement in neurological functions, including ambulation, manipulation, language, and swallowing, was reported in 6/8 patients with late-infantile onset and 1/3 with juvenile onset, compared with 1/8 patients with early-infantile onset. In contrast, early-infantile onset patients showed limited sustained benefit, despite earlier intervention in some. Gastrointestinal events were the predominant treatment-related AEs, and three participants discontinued miglustat because of AEs. Three deaths occurred from NPC2-associated liver failure, respiratory failure, and aspiration pneumonia; none were attributed to miglustat [4].

Fecarotta et al. [1] led an Italian multicenter open-label study, enrolling 25 NPC patients ranging in age from 7 months to 44 years, who received miglustat for 48 to 96 months. At 24 months, most evaluable participants showed improvement or stabilization across several neurological domains. For swallowing, improvement or stabilization was reported for water in 65%, purée in 58%, small pasta in 60%, and biscuits in 55% of 20 evaluable patients; these findings persisted in approximately 40–50% during the 48–96-month follow-up for most consistencies. Long-term stabilization or improvement in the Mean Composite Severity Score (MCSS) was observed principally in juvenile- and adult-onset disease. Among the seven patients who initiated miglustat within 3.5 years of neurological onset, 4/7 (57%) improved or stabilized, whereas improvement or stabilization was uncommon after later initiation, occurring in only one later-treated patient. The safety profile aligned with previous findings, with diarrhea and weight loss being the most common side effects. One participant was hospitalized for uncontrolled seizures, which the investigators attributed to NPC rather than miglustat; no reported AE was considered life-threatening [1].

Zhang et al. conducted a prospective, single-arm phase IV study in China that involved 17 participants. They were aged 6–33 years and received miglustat for 52 weeks; 14 completed treatment and 13 had evaluable primary-outcome data. From baseline to Week 52, mean saccadic peak acceleration increased by 19.2% (95% CI 11.7–26.7), mean saccadic velocity increased by 12.5%, mean peak duration decreased by 6.5%, and the linear regression slope decreased by 15.6%. The ocular-movement component of the Pineda Disability Scale improved numerically by 0.4 points, while ambulation, manipulation, language, swallowing, and seizure domains generally showed smaller changes or stabilization. All 17 participants experienced at least one treatment-emergent adverse event (TEAE), and treatment-related TEAEs occurred in 12; diarrhea was the most frequent event (12/17). Five participants experienced SAEs. Two fatal events, accidental death and asphyxia, were considered unrelated to miglustat. As this study had no comparator, the observed oculomotor changes could not be separated from natural variation, concomitant treatment, or other time-related effects [30,31].

Across the six miglustat studies, findings were more consistent with stabilization or slower deterioration in selected neurological domains than with recovery of established impairment. The controlled evidence was limited by the non-significant prespecified primary analysis, while the longer-term evidence arose from uncontrolled and partly overlapping cohorts. Gastrointestinal AEs, particularly diarrhea, flatulence, abdominal discomfort, and weight loss, were consistently reported but were generally described as manageable or decreasing during continued treatment. The studies retrieved from the databases for miglustat are summarized in Table 1. 

#### 3.4.2. Arimoclomol

Arimoclomol, an HSP amplifier, was investigated in a multinational interventional study involving patients with NPC. In a two-part phase 2/3 clinical trial by Mengel et al., participants were randomized 2:1 to receive either arimoclomol (n = 34) or placebo (n = 16), administered three times daily for 12 months. Patients receiving miglustat could participate if they had been receiving a stable dose for at least six months before enrollment. At the 12-month endpoint, the average NPCCSS score had slightly worsened by 0.76 points in the arimoclomol group, indicating slight worsening of disease symptoms, compared to 2.15 points in the placebo group, showing more rapid worsening of disease symptoms. This translated to a least squares mean treatment difference of −1.40 points (95% CI: −2.76 to −0.03; *p* = 0.046), corresponding to a significant treatment effect in favor of arimoclomol and corresponding to a 65% relative reduction in the rate of disease progression. Principal secondary outcomes did not consistently separate the groups: 5DNPCCSS responder rates were 50.0% with arimoclomol and 37.5% with placebo (*p* = 0.546), and Clinical Global Impression of Improvement responder rates were 58.8% and 56.3%, respectively (*p* = 1.000). Other secondary measures, including time to or proportion of worsening, the 17-domain NPCCSS, SARA, 9-Hole Peg Test, EQ-5D-3L-Y, and NPC clinical database score, were not statistically different between groups [10].

Clinically homogeneous populations were grouped together for subgroup analyses, and this showed enhanced treatment effects of arimoclomol. Among those aged 4 and older (n = 44), the mean difference in the 5-domain NPCCSS score at 12 months favored arimoclomol over placebo by −1.80 (95% CI: −3.24 to −0.35; *p* = 0.016), reflecting an 82% reduction in disease progression. A similar benefit was observed in patients receiving miglustat (n = 39), with a mean difference of −2.06 (95% CI: −3.49 to −0.63; *p* = 0.006), suggesting disease stabilization. In contrast, due to the small sample size and baseline imbalances, there was no meaningful difference seen in those not receiving miglustat (n = 11; *p* = 0.284) or in children under 4. When patients with two non-functional NPC1 mutations were excluded (n = 47), the treatment difference was −1.61 (*p* = 0.020). Similarly, another analysis focused on patients with an Annual Severity Increment Score (ASIS) between 0.5 and 2.0 (n = 21) showed a trend favoring arimoclomol (−2.39), though it did not reach statistical significance (*p* = 0.054) [10].

Exploratory biomarker analyses showed a mean increase in HSP70 of 1778.98 pg/mL with arimoclomol at 12 months (*p* = 0.001) and lower lysosphingomyelin-509 levels than with placebo (*p* = 0.043). Numerical reductions in serum cholestane-triol (−5.50 ng/mL; *p* = 0.225) and unesterified cholesterol in peripheral blood mononuclear cells (−44.44 μg/mg protein; *p* = 0.096) were not statistically significant. AEs were common but mostly mild to moderate. During the double-blind phase, 88.2% of arimoclomol-treated patients reported AEs, compared to 75.0% on placebo. SAEs occurred less often with arimoclomol (14.7%) than placebo (31.3%). Notable TEAEs included vomiting, urticaria and angioedema. One patient on arimoclomol died after experiencing cardiorespiratory arrest (2.94%), but this was related to NPC progression [10].

The 48-month open-label extension enrolled 41 participants from the parent trial, of whom 29 completed 48 months. Among completers, the mean increase in 5DNPCCSS was 3.2 ± 4.8 points over 48 months, equivalent to approximately 0.8 points per year; the corresponding increase in the rescored 4-domain NPCCSS was 2.7 ± 4.2 points, or approximately 0.7 points per year. Among participants who crossed from placebo to arimoclomol, the annual 5DNPCCSS progression rate decreased from 2.0 points during placebo exposure to 0.1 points during the first year of arimoclomol. The rescored 4-domain progression rate similarly decreased from 1.9 to 0.2 points per year. These within-participant findings were directionally consistent with the randomized trial but were obtained after removal of the concurrent placebo comparison. During the extension, 92.7% of participants experienced an AE and 36.6% experienced an SAE. Moderate proteinuria in one participant was considered a treatment-related SAE. Two deaths occurred and were considered unrelated to arimoclomol [17].

The open-label infant substudy enrolled five participants aged 14–23 months at screening, all of whom also received miglustat. Arimoclomol exposure ranged from 72 to 1109 days, and four infants remained in the study for more than 12 months. Changes in Bayley Scales of Infant and Toddler Development, Third Edition (BSID-III) scores and biomarkers varied between individuals and did not show a consistent developmental efficacy pattern. All five infants experienced AEs, with 108 events reported overall; 15 SAEs occurred in two participants and were considered unrelated to arimoclomol. Elevated alanine and aspartate aminotransferase levels in one participant were considered probably treatment-related, resolved after treatment interruption, and resulted in withdrawal. No deaths occurred. The substudy therefore principally extended pharmacokinetic and safety observations to children younger than 2 years [18].

Overall, the randomized trial demonstrated a statistically significant difference in the primary 5DNPCCSS outcome, particularly in participants receiving concomitant miglustat, but most secondary clinical outcomes were not statistically significant. The extension findings were directionally consistent with slower measured progression during arimoclomol exposure, although the uncontrolled design, attrition, and reuse of the parent trial cohort limited independent confirmation. The studies retrieved from the databases for arimoclomol are summarized in Table 2. 

#### 3.4.3. NALL

The search for treatment options that could offer symptomatic relief and functional stabilization for NPC beyond substrate reduction has led to the development of the compound NALL, which is a modified amino acid with neuroprotective properties. Three study reports evaluated NALL: a short-term open-label, rater-blinded phase II study; a randomized placebo-controlled crossover phase III trial; and a long-term open-label extension using an external natural-history comparison [12].

The open-label study, conducted by Bremova-Ertl et al., was a rater-blinded, multinational Phase II trial that assessed NALL’s short-term symptomatic effects. The study enrolled 33 genetically confirmed NPC patients across five countries, ranging in age from 7 to 64, with over six weeks of treatment followed by a six-week washout period. Patients aged ≥13 years received 4 g per day, while younger children were given adjusted doses based on body weight. Those already taking miglustat could stay on it if they had been on a stable dose for at least six weeks before starting NALL. The primary outcome was the CI-CS, which is a novel video-based assessment where blinded reviewers compared patient performance on either an 8MWT or a 9-Hole Peg Test. NALL showed a significant positive effect, with a mean improvement of 0.86 points on the CI-CS scale (*p* = 0.029). Most patients improved during treatment, and then worsened again during the washout period, suggesting a treatment-associated effect, although placebo and learning effects cannot be completely excluded. The secondary outcomes also supported these findings. SARA improved by 1.19 points during treatment (*p* = 0.001) and worsened by 1.45 points during washout (*p* = 0.002). Investigator-, caregiver-, and patient-reported Clinical Global Impression of Change (CGI-C) scores all showed similar patterns, which showed improvement during treatment and decline afterward. Other measures like the mDRS and SCAFI also leaned in a favorable direction, though the changes were not statistically significant. QoL, as measured by the EQ-5D, stayed about the same. NALL was generally well-tolerated. No SAEs were tied to the treatment. Four patients reported a total of seven TEAEs, including flatulence, diarrhea, increased hunger, rash, and restlessness with aggressive behavior. Mild issues like headaches and colds were the most common unrelated side effects. One patient dropped out due to unrelated seizures, and another withdrew after an accidental fall [8].

Building on these findings, a randomized, double-blind, placebo-controlled, crossover Phase III study further assessed NALL’s efficacy in patients with NPC. Also conducted by Bremova-Ertl et al., this trial enrolled 60 symptomatic individuals aged ≥4 years with genetically or clinically confirmed diagnoses. Participants received NALL and placebo for two consecutive 12-week treatment periods, with an immediate switch between treatments. The primary endpoint outside the U.S. was the change in total SARA score, while in the U.S., the modified SARA (mSARA) was used in line with FDA guidance. The mDRS was a secondary endpoint. The mean change in SARA score was −1.97 ± 2.43 with NALL versus −0.60 ± 2.39 with placebo, a least-squares mean difference of −1.28 points (95% CI: −1.91 to −0.65; *p* < 0.001). The mDRS also showed a significant benefit, with a mean difference of −0.029 (95% CI: −0.048 to −0.010), indicating improved overall function. Key secondary outcomes supported these results. The investigator-rated CGI-I showed a –0.6 point difference (95% CI: −1.1 to −0.1), and the caregiver-rated CGI-I showed a −0.7 point difference (95% CI: −1.2 to −0.2), both favoring NALL. The exploratory NPC Clinical Severity Scale difference was −0.5 points (95% CI: −1.2 to 0.2) and was not statistically significant. NALL was well tolerated, with AEs reported in 36 patients during NALL treatment (79 events) and in 30 patients during placebo (75 events). No serious TEAEs occurred. Mild, transient side effects associated with NALL included anal incontinence, restless legs, and rosacea [12].

The open-label extension enrolled 53 participants from the phase III trial, of whom 49 formed the modified intention-to-treat analysis. At 12 months, mean change in the 5-domain NPC Clinical Severity Scale was −0.27 ± 2.42 points during NALL treatment, compared with an expected increase of 1.50 ± 3.16 points derived from an external natural-history cohort; the mean difference was −1.77 points (95% CI −3.05 to −0.48; *p* = 0.009). At 18 months, the corresponding changes were +0.05 ± 2.95 and +2.25 ± 4.74 points, respectively, producing a difference of −2.20 points (95% CI −4.06 to −0.35; *p* = 0.023). Mean SARA scores remained below the parent-trial baseline at both 12 and 18 months. No AE led to discontinuation, and no treatment-related SAE or death was reported. Although the extension findings suggested maintenance of neurological outcomes, the external historical comparison provided weaker causal evidence than the randomized crossover phase [19].

Across the three studies, NALL demonstrated a reproducible short-term effect on ataxia and functional outcomes, supported by the randomized crossover trial. The extension showed persistence of the observed pattern during longer exposure, but its single-arm design and reliance on an external natural-history cohort did not independently establish a disease-modifying effect. The studies retrieved from the databases for NALL are summarized in Table 3. 

#### 3.4.4. HP*β*CD

To date, eight studies have analyzed the safety and efficacy of HPβCD administered intrathecally or intravenously in patients with NPC. These studies have helped expand the growing body of evidence that HPβCD might help in slowing down the progression of NPC neurological symptoms, particularly those affecting cognition and motor function.

An open-label phase I/IIa trial conducted in 2017 by Ory and colleagues investigated HPβCD via intrathecal administration in 14 neurologically symptomatic patients at the NIH, along with three more patients receiving biweekly dosing at Rush University. The primary objective was to assess safety and tolerability. No SAEs were associated with the drug itself, but it was noted that all NIH participants developed new or worsening mid-to-high frequency hearing loss, which was managed with hearing aids. Other common side effects included post-lumbar puncture headaches, which were reported in about 64% of participants, and dose-related fatigue. Development of hepatocellular carcinoma was seen in one patient, but it was attributable to pre-existing disease rather than the treatment. Despite the auditory impairments, the trial showed encouraging signs of efficacy. Patients treated with HPβCD had a significantly slower rate of neurological decline compared to historical controls, as measured by the annual change in total NSS (1.22 vs. 2.92 points per year; *p* = 0.0002). This remained true even when the hearing domain was excluded (0.69 vs. 2.67; *p* < 0.0001). Significant improvements were also seen in cognition (*p* = 0.0040) and speech (*p* = 0.0423), with a trend toward better ambulation (*p* = 0.0622). As expected with an ototoxic drug, hearing continued to decline (*p* = 0.0518). These clinical findings were backed by biomarker data. Plasma and CSF levels of 24(S)-hydroxycholesterol rose after the treatment, indicating biological activity. FABP3 and calbindin D, which are markers of neuronal damage in the CSF, declined significantly, which suggested potential neuroprotective effects. In patients who also received miglustat, benefits in ambulation (*p* = 0.0223), cognition (*p* = 0.0061), and speech (*p* = 0.0218) remained significant [13].

Following the completion of the Phase I/IIa trial of intrathecal HPβCD, a long-term open-label continuation study led by Farmer et al. was conducted in 2019. This study tracked the neuropsychological outcomes in children and adolescents with NPC1 over 36 months. Fourteen participants, ages 4 to 23, were repeatedly assessed for cognitive function, adaptive behavior, and memory to gauge the long-term impact of treatment. On average, FSIQ declined by 1.28 points per year (SE = 0.70), a change that did not reach statistical significance (*p* = 0.076), suggesting that most participants maintained relatively stable cognitive function. In contrast, the Vineland-II Adaptive Behavior Composite score dropped significantly by 1.76 points per year (SE = 0.67; *p* = 0.011). While standard scores fell, the individual domains, including communication, socialization, daily living skills, and motor skills, all showed upward trends over time. However, none of them were statistically significant (all *p* > 0.1). These results indicate that the individuals continued to improve their skills over time, but their progress was slower than what would typically be expected for their age. Immediate, delayed, and recognition memory measures remained broadly stable during the reported follow-up. AEs were not systematically tracked in this follow-up study. Still, 79% of participants had hearing loss at the start, likely due to the known ototoxic effects of intrathecal HPβCD seen in earlier research. No new safety concerns were reported during the continuation period [20]. As the same cohort was followed without a concurrent comparator, this publication extended outcome duration but did not provide independent efficacy confirmation.

The Phase IIb/III randomized, double-blind, sham-controlled trial was conducted by Mandos LLC. (Thousand Oaks, CA, USA). Patients selected were aged between 4 and 21 years with neurological symptoms of NPC. Fifty-six participants were randomized to receive either VTS-270 (n = 38) or a sham procedure (n = 18) over a 52-week period. The main objective was the change in a 4-domain composite NPC Severity Score (NPC-SS) at Week 52, which showed no statistically significant difference between the two groups. Exploratory measures including CGIC score distributions, domain-specific changes in NPC-SS (with and without hearing adjustments), responder analyses, QoL scores (EQ-5D-3L VAS), time to 1-point worsening, and functional tests like the Timed Up and Go and 9-Hole Peg Test also did not show consistent or meaningful clinical benefits. Biochemical markers declined slightly but not significantly. Two participants died during the trial, one in the VTS-270 group during Part A (33.3%) and one in the sham group during Part B (6.7%). SAEs included lymphadenitis and deafness [24].

After the initial randomized, sham-controlled trial of VTS-270 [24], Mandos LLC launched an open-label, single-arm extension study (Part C). This study aimed to track the long-term safety of intrathecal VTS-270 in patients with neurologic symptoms of NPC. A total of 66 participants were enrolled, ranging in age from 4 to 21, including both treatment-naive patients and those from earlier trial phases. Doses were given every two weeks, with protocol changes approved by both investigators and the sponsor. TEAEs were reported in nearly all participants: 100% of treatment-naive and Phase I patients and around 97.1% of those from Parts A and B suffered from them. SAEs such as tachycardia and different types of hearing loss occurred in about half of the group. In the extension phase, two participants died. One was from the treatment-naive group, and the other had taken part in earlier phases. Despite the high rate of AEs, the overall safety profile stayed in line with previous findings. No new safety risks were identified. No new safety category was identified, but the rollover design and absence of a concurrent control meant that this record primarily contributed longer-term safety rather than independent efficacy evidence [25].

Two pediatric patients under 18 who had previously completed Part B of the Phase 2b/3 VTS-270 [25] joined an open-label, single-arm extension study. These patients had previously discontinued treatment in earlier trial phases due to AEs. During the follow-up period of about 156 weeks, both patients reported only two TEAEs in total, of which none of them were considered serious. No deaths occurred during the study. After thorough clinical evaluations and risk assessments of the patients, they were approved to resume therapy under close medical supervision. No new safety issues or unexpected side effects emerged throughout the extended treatment. These findings support the feasibility and tolerability of long-term VTS-270 use in carefully monitored pediatric patients with neurologic symptoms of NPC1 [26].

Building on the same primary outcomes as the earlier pediatric extension study, another interventional open-label trial evaluated the safety and tolerability of intrathecal Adrabetadex (HPβCD) in 27 patients. The patients were aged 16 to 21 years with neurologic manifestations of NPC type C. The cohort included 16 males and 11 females, comprising both treatment-naive individuals and those previously treated with Adrabetadex. Over a follow-up period extending up to 134 weeks, TEAEs were reported in all treatment-naive patients (9/9) and in 94.4% (17/18) of previously treated participants. SAEs occurred in 55.6% of treatment-naive patients and 50.0% of previously treated patients. Reported SAEs included ventricular extrasystoles, deafness, and constipation. Despite the frequency of AEs, no new safety signals emerged, and the safety profile remained consistent with prior studies [27].

In 2022, Hastings et al. worked on the same drug but differed in the route of administration. In comparison to the other studies [13,20,24,25,26,27] that used intrathecal administration of HPβCD, this study used intravenous administration. Over a period of 14 weeks, 13 adult patients received biweekly IV infusions of either 1500 mg/kg or 2500 mg/kg. The primary outcomes assessed safety, tolerability, and pharmacokinetics, all of which showed an overall acceptable safety profile. Treatment was discontinued by three participants. One participant discontinued due to hypersensitivity pneumonitis and two due to transient hearing loss. No new safety concerns were identified. Pharmacokinetic data showed a plasma half-life of roughly two hours, with low but persistent CSF concentrations (~33 μM), lasting longer than in plasma. Secondary outcomes indicated both peripheral and central pharmacodynamic effects as serum lathosterol levels dropped, 4β-hydroxycholesterol increased, PPCS and liver filipin staining decreased, CSF total Tau levels declined in 8 of 10 patients, and serum 24(S)-hydroxycholesterol rose after infusion. Clinical severity scores (17D-NPC-CSS) remained stable, with some participants noting improvements in swallowing, speech, gait, and cognitive engagement [6].

Sharma et al. conducted a 48-week intravenous phase I/II study in which 12 participants were randomized among active-dose groups receiving 1500, 2000, or 2500 mg/kg every two weeks. Nine participants completed 48 weeks, and 8/9 met the primary responder definition of a ≥1-point improvement in at least two domains of the 17-domain NPC Clinical Severity Scale. Clinicians rated 7/9 participants as improved and 2/9 as stable, while all nine were rated as improved or stable by Patient Global Impression of Change. Serum 24(S)-hydroxycholesterol peaked 22% above baseline after the first infusion, and PPCS decreased by 61.3% at Week 16 in the limited evaluable biomarker sample. All 12 participants experienced at least one TEAE, and 5/12 experienced an SAE; no death or TEAE-related withdrawal occurred. Slight-to-mild hearing loss occurred in two participants and transient hearing changes in three. As all groups received active treatment and the clinical analyses were descriptive, the responder proportion could not be compared with untreated disease [21].

Across the HPβCD studies, pharmacodynamic target engagement was observed with both intrathecal and intravenous administration. Clinical efficacy findings were inconsistent: favorable historical-control and uncontrolled observations contrasted with the absence of benefit in the sham-controlled trial. Hearing impairment was the most consistent treatment-associated safety finding, particularly after intrathecal administration; the frequency and reversibility of hearing changes following intravenous administration remained uncertain because of the small cohorts. The studies retrieved from the databases for HPβCD are summarized in Table 4 and Table 7. 

#### 3.4.5. Efavirenz

Gascón-Bayarri et al. conducted a phase II, open-label study of low-dose efavirenz added to standard care, including miglustat, in 16 participants with late-juvenile- or adult-onset NPC and cognitive impairment. Efavirenz was administered at 25 mg/day for the first 26 weeks and increased to 100 mg/day for Weeks 27–52. At Week 52, all 16 participants met the prespecified primary responder criterion, defined as absence of deterioration in at least two of three cognitive domains: dementia severity, verbal memory, and executive function. This endpoint represented multidomain non-deterioration rather than improvement in every cognitive measure; individual domain results included both improvement and worsening among participants [22].

Post hoc classification identified seven participants with a more favorable pattern and nine with a less favorable pattern; shorter duration since neurological onset was observed in the more favorable group. These subgroups were derived after observing the data rather than defined through randomization, and all nine participants in the less favorable subgroup still fulfilled the prespecified primary response definition. Three AEs occurred in one participant and were judged unrelated to efavirenz; no treatment-related SAE was reported. The study demonstrated feasibility and 52-week cognitive stability according to its responder definition, but the single-arm design and continuation of background therapy prevented isolation of the efavirenz effect [22]. The studies retrieved from the databases for Efavirenz are summarized in Table 5.

#### 3.4.6. Nizubaglustat

The phase II RAINBOW study evaluated nizubaglustat in a mixed cohort of 13 participants: seven with GM2 gangliosidoses and six with NPC. Participants were randomized to 3 mg, 9 mg, or placebo for 12 weeks and could subsequently enter an active-treatment extension. The primary outcomes were pharmacokinetics, pharmacodynamics, and safety rather than clinical efficacy. Nizubaglustat was rapidly absorbed and produced dose-dependent reductions in plasma C16 and C18 glucosylceramides, with greater target engagement at 9 mg. No formal clinical-efficacy hypothesis test was undertaken during the initial 12-week period [23].

Twelve participants entered the extension, and 11 remained in the reported analysis, including four participants with NPC. Across the combined GM2 and NPC extension cohort, 45.5% improved by ≥1 point or remained stable on SARA, 54.6% improved or remained stable on functional SARA, and 63.7% improved or remained stable on the Inventory of Non-Ataxia Signs. These outcomes were not reported separately for the NPC subgroup. During the initial 12 weeks, 10/13 participants experienced TEAEs, all mild or moderate, with no SAE or treatment discontinuation. One unrelated liver abscess occurred during the extension. The study demonstrated brain-penetrant pharmacodynamic activity and short-term tolerability, but the small NPC subgroup and combined-diagnosis reporting prevented conclusions about clinical efficacy specifically in NPC. [23] The studies retrieved from the databases for nizubaglustat are summarized in Table 6.

#### 3.4.7. Vorinostat

An evolving treatment therapy is the HDAC inhibitor called Vorinostat. It was tested in a Phase I/II open-label trial with patients with genetically confirmed NPC1. Twelve participants took part in this study. For three days each week, they took the medicine and stopped for the next four days. They initially started with a daily dose of 200 mg, which was later increased to 400 mg for the last three months of the six-month study. The drug demonstrated acceptable tolerability, as all participants were able to complete the lower dose phase of the study, and 91.7% made it through the higher dose phase. Serum levels of CatD and galectin-3 (LGALS3) were also monitored in the study and exhibited slight decreases, which were not statistically significant but remained above normal levels. Until this point, vorinostat appears to be a promising candidate, but the safety profile raises concerns. Although there were no deaths, serious side effects, including vomiting, Epstein–Barr viraemia, and fever, were observed in 41.7% of subjects. Because the study primarily assessed safety, tolerability, and biochemical endpoints and included neither a comparator nor a clinical efficacy endpoint, it did not establish neurological benefit [28]. The studies retrieved from the databases for vorinostat are summarized in Table 7.

#### 3.4.8. N-Acetylcysteine (NAC)

A different approach for the treatment of NPC is targeting oxidative stress. NAC, a well-known antioxidant, was investigated in a phase 1/2 crossover trial to see if it was able to lower oxidative damage that occurred in patients suffering from NPC. Thirty-five participants received NAC and a placebo in alternating eight-week phases, with washout periods in between. The primary outcome measured in this study was levels of oxysterols, a biomarker of oxidative stress, as they are a product and driver of oxidative stress. The oxysterol levels ranged from 28.09 ng/mL with NAC and 27.64 ng/mL with placebo. The difference between the results was minimal. SAEs were reported according to randomized sequence rather than the treatment phase in which they occurred: 3/17 participants in the placebo-to-NAC sequence and 5/18 in the NAC-to-placebo sequence experienced an SAE. Treatment-specific attribution was therefore not possible. The available controlled evidence did not demonstrate a biochemical benefit of NAC [29]. The studies retrieved from the databases for NAC are summarized in Table 7.

Overall, statistically significant controlled effects were demonstrated for the primary 5DNPCCSS outcome with arimoclomol and for SARA with NALL. The prespecified primary miglustat analysis did not reach statistical significance, and the sham-controlled intrathecal Adrabetadex trial did not demonstrate separation from sham despite favorable historical-control findings in the earlier HPβCD program. Efavirenz, nizubaglustat, and intravenous HPβCD provided preliminary evidence of clinical stability or pharmacodynamic activity, but their efficacy findings were constrained by uncontrolled designs, active-dose-only comparisons, combined-diagnosis analyses, or small samples. Extension studies added information on durability and safety but generally did not provide independent controlled confirmation of efficacy.

## 4. Discussion

This systematic review collated evidence from 24 studies involving 475 participants, accounting for any extension studies. The findings suggest that miglustat, arimoclomol and NALL showed the most consistent stabilization or improvement in neurological function. On the other hand, HP*β*CD, while demonstrating biological activity also displayed inconsistent clinical findings, and significant safety concerns. The available studies for NAC and vorinostat were not found to have significant conclusions on their efficacy and safety. Meanwhile, the evidence for efavirenz and nizubaglustat remains preliminary. Due to the rare status of the disease, the included studies have a limited number of participants, so these outcomes and conclusions must be interpreted within this context. It is also important to note that although stabilization in neurological function is key, changes that occur without a comparator cannot automatically be attributed to the treatment.

Miglustat was the first medicine approved in Europe for NPC; levacetylleucine was also authorized in the EU on 19 January 2026. Across six studies, it was found to show stabilization in neurological manifestations in domains like swallowing, ambulation, horizontal saccadic eye movement (HSEM) and cognition [1,2,3,4,16]. However, in the complete analysis of a pivotal RCT, the primary HSEM-**α** comparison did not obtain statistical significance; it only gained statistical significance when a sensitivity analysis was conducted that excluded participants on benzodiazepines. Though several secondary outcomes were not statistically significant, there was marked improvement in swallowing and slower deterioration in ambulation. The open-label and pediatric cohort studies demonstrated consistent stabilization in disease progression, particularly in late-infantile, juvenile, and later-onset patients, though early infantile disease progressed despite treatment. Therefore, better outcomes appeared with early initiation of treatment, and onset of the disease seems to play a pertinent role in the same [4]. These conclusions were mirrored by other systematic reviews and meta-analyses conducted on the relationship between NPC and miglustat [32]. A systematic review by Santos-Lozano reported that miglustat was able to delay neurodegeneration, particularly in patients with late-onset disease. The included prospective Chinese phase IV study extended these findings to an East Asian population. Several HSEM parameters improved by week 52, though the Pineda Disability Scale demonstrated small improvements or stabilization in ocular movement, swallowing, language, manipulation and seizure domains. Despite these findings, since the study does not have a comparator and analyses were based on small numbers, the study only supports the potential use of miglustat in NPC patients, but it cannot confirm its efficacy. AEs were manageable despite being common with the use of miglustat—gastrointestinal AEs in particular. Any fatal events were reportedly due to accidents, asphyxia, or advanced NPC complications, and could not be directly attributed to miglustat. Therefore, they should not be interpreted as treatment-related mortality [33]. Three observational studies did not meet our eligibility criteria but had significant related results. Miglustat was associated with greater preservation of swallowing, as well as a lower aspiration risk among 50 patients with neurologically symptomatic NPC, as found by the study conducted by Solomon et al. [34]. Patterson et al.’s final NPC Registry report found there was stabilization or improvement of three of four disability domains in 70.5% of continuously treated patients [35]. A pooled retrospective analysis of 789 patients found lower mortality with exposure to miglustat when survival was measured from neurological onset and from diagnosis [36]. Though these studies increase the support for miglustat use in NPC, they remain observational studies. Many factors prevent these findings from being absolutely attributable: disease severity, treatment selection, access to specialist care, survivor bias, and overlap between registry populations. However, together, the randomized and observational studies support the use of miglustat as a treatment that may slow the progression of the disease as opposed to allowing full neurological recovery.

Miglustat has recently been accompanied in its approval status by Arimoclomol as of 2024, to be taken in conjunction with miglustat [37]. Arimoclomol was evaluated through a randomized phase 2/3 trial as well as two extensions with the same clinical development program. Progression on the 5-domain NPCCSS was found to be slower with arimoclomol compared to the placebo in the randomized phase, with an adjusted treatment difference of −1.40 points. The confidence interval approached the null value, and several secondary outcomes did not have a significant difference, with the major treatment effect being observed among participants on concomitant miglustat being derived from a subgroup analysis [10]. The following post hoc analysis using the 4-domain NPCCSS also preferred arimoclomol, with a treatment difference of −1.70 points [17]. A 48-month open-label extension demonstrated that progression slowed, as opposed to being completely halted, supported by the mean 5- and rescored 4-domain NPCCSS increasing by 3.2 and 2.7 points, respectively. Patients who were previously taking placebo but switched to arimoclomol had a mean annual 5-domain NPCCSS change decrease from 2.0 to 0.1 in the first year taking arimoclomol. However, since the comparison is occurring within each patient’s own parameters as opposed to having a concurrent placebo arm, it makes the support suggestive rather than definitive [17]. An open-label infant substudy was conducted, giving further information on the safety and tolerability, but it included only five infants, all of whom received miglustat concomitantly. Responses varied between the participants, with one participant withdrawing due to elevations in ALT and AST. The study therefore supports the feasibility of administering arimoclomol before two years of age, but not efficacy, particularly since the study population is small, all the participants were also on miglustat, and there was no control group [18]. The proposed mechanism of action in providing this benefit is that arimoclomol, beyond its HSP70 chaperone effects, might also act through TFEB signaling and lysosomal stabilization. TFEB is a transcription factor that governs lysosomal biogenesis and autophagy, which is relevant in NPC as it is a lysosomal storage disorder. Despite this, the biological link is suggested and cannot be entirely proven to be the cause of these effects [11].

The included studies demonstrated that NALL showed a rapid symptomatic response in patients, particularly in neurological function. The rater-blinded open-label study showed improvement when patients were taking NALL, and subsequent decline in their neurological function during washout [8]. On the other hand, the randomized, double-blind crossover trial showed significant benefits on SARA and mDRS, as well as clinician- and caregiver-rated global outcomes [12]. This demonstrates that NALL is effective for symptomatic treatment, but it cannot definitively be said that NALL has any long-term effect since the treatment period was only 12 weeks. An included open-label extension following 53 participants from the randomized NALL trial showed that the mean 5-domain NPCCSS scores remained approximately stable after 12 and 18 months, and improvement in SARA that was present in the randomized phase was maintained. No treatment-related SAEs or deaths were reported [19]. Though the findings are encouraging, several factors act against these supportive findings: the open-label design, the non-independent study population, and comparison with unmatched historical progression estimates. Therefore, there is significant evidence that NALL is effective in short-term symptomatic treatment; long-term disease-modifying action still needs confirmation through controlled follow-up.

Uncontrolled intrathecal studies for HP*β*CD demonstrated slower neurological progression compared to historical controls. Historical control efficacy analysis was post hoc, and participants were not randomly assigned to HP*β*CD or natural-history follow-up. In comparison, the larger randomized, double-blind, sham-controlled, Adrabetadex trial did not show any major benefit on its primary 4-domain NPC severity endpoint, and it did not demonstrate consistent benefit in exploratory outcomes. This discrepancy between the positive uncontrolled and the controlled trials may reflect the multiple factors involved rather than specifically the treatment effect. Therefore, the controlled result should be used to judge the clinical efficacy. Two active-dose-ranging studies were conducted for IV HP*β*CD. The 14-week study demonstrated systemic and central pharmacodynamic activity. The 48-week study reported that all 9 participants either improved or were stable [6,21]. Participants were not randomized to HP*β*CD versus placebo or no treatment, meaning that the trials can compare doses, but they cannot effectively prove improvement. In the 48-week trial specifically, only 9 out of the initial 12 completed treatment; efficacy analysis was descriptive, and the responder definition allowed a participant to qualify based on selected areas rather than showing improvement in overall disease severity. The studies justify further controlled investigation, but they do not resolve the negative findings from the sham-controlled intrathecal trial conducted. Despite the possibility of improving disease progression, HP*β*CD had some significant AEs. Hearing impairment in particular remains the most consistent treatment-associated AE, particularly with intrathecal administration, where mid-to-high-frequency hearing loss was reported frequently. Though hearing changes occurred with IV administration as well, it was less consistent [6,13,20,24,25,26,27]. It is also important to note the possible effects of repeated lumbar punctures as well as other possible disease-related or procedure-related AEs which could outweigh the benefits of the medication. Therefore, although HP*β*CD can have a positive effect, it does not demonstrate a consistent benefit sufficient to outweigh the possible negative outcomes.

Efavirenz works by enhancing neuronal cholesterol turnover through cholesterol 24-hydroxylase (CYP46A1). A 52-week, single-arm study was conducted including 16 participants, all of whom met the primary responder criterion. It is important to note that this was defined as the absence of deterioration in a minimum of two out of the three cognitive domains, rather than the patient improving in all measures. Quantitative testing was conducted and showed a mixture of stabilized and worsening cognitive functions, with the favorable and unfavorable subgroups being created post hoc. In addition, all participants were receiving standard care with miglustat, making it difficult to isolate the effect of efavirenz. No efavirenz-related AEs or SAEs were reported, which suggests that it is a safe drug [22]. The results support a controlled trial, but they are not reliable enough to establish efavirenz as an effective disease-modifying therapy in NPC.

Nizubaglustat is a brain-penetrant azasugar that targets glucosylceramide metabolism. The RAINBOW study included 13 participants, seven of which were with GM2 gangliosidoses and six with NPC. The 12-week randomized and controlled period showed that nizubaglustat produced dose-dependent reductions in plasma C16 and C18 glucosylceramides, was tolerated well, and supported biological target engagement. The extension lacked a concurrent control group since the placebo group was converted to receive active treatment. At the point of analysis, only 4 NPC patients remained, and many of the reported findings were grouped together with GM2 gangliosidoses patients [23]. Therefore, the study cannot be reliably utilized to estimate the efficacy of nizubaglustat in NPC. An extension was proposed for patients who have completed the 12-week period of treatment. This could give a deeper insight into the efficacy of nizubaglustat in the management of NPC, particularly in a long-term setting [38]. An overlapping open-label study is also currently being conducted, including patients who completed the RAINBOW trial, as well as 10 patients who received a full dose of miglustat for 12 months with stable or worsening condition who now wish to switch to nizubaglustat. The main aim of this study is to evaluate the long-term tolerability, safety, pharmacokinetics, and efficacy of the drug [39]. Nizubaglustat is also being assessed in another ongoing trial: an 18-month double-blind, randomized, placebo-controlled, multicenter, Phase 3 study aiming to evaluate the safety and efficacy of oral nizubaglustat in late-infantile and juvenile NPC as well as GM1 and GM2 gangliosidoses [40].

Vorinostat was assessed in a small, open-label phase 1/2 study involving 12 participants. All 12 completed the 200 mg phase, and 11 completed the 400 mg phase, though no convincing efficacy results were reported. Although five patients experienced SAEs, a causal relationship could not be established between the AEs and vorinostat [28]. Therefore, the available evidence is insufficient to support therapeutic efficacy, despite establishing short-term feasibility.

In a similar vein, the randomized crossover trial of NAC did not show a meaningful decrease in the primary oxysterol outcome as compared with the placebo. NAC was generally tolerated, but some SAEs were reported in a randomized sequence, making it difficult to attribute them to NAC or placebo [29]. Rather than relying only on the theoretical antioxidant activity, future trials would need stronger dose rationale, verified central nervous system (CNS) exposure, and clinically relevant outcomes.

Developing reliable biomarkers is also vital in NPC in order to better detect neurological decline. Validated biomarkers could help identify the disease earlier, support dose selection, and monitor progression. Reliable biomarkers may also complement clinical endpoints by providing objective measures of pharmacodynamic target engagement and treatment response, particularly in clinical trials where changes in clinical severity may be slow or difficult to detect. Biomarkers that change before overt clinical deterioration could facilitate earlier assessment of therapeutic activity and improve trial design. Of the particular biomarkers involved, 24(S)-hydroxycholesterol [24(S)-HC] is particularly relevant since it is generated from cholesterol in the brain by CYP46A1 and can reflect cholesterol turnover. Both IV and intrathecal HP*β*CD studies reported a transient increase in 24(S)-HC after administration, a finding that is consistent with the mobilization and metabolism of previously sequestered cholesterol [6,13]. The 48-week IV study also showed an early rise in 24(S)-HC with a reduction in PPCS. Though these biomarker changes demonstrate pharmacodynamic target engagement, they should not be considered as validated surrogate markers of clinical benefit. A post-dose increase in 24(S)-HC does not prove that neurological decline has slowed, as evidenced by the inconsistency between biological activity and controlled clinical efficacy findings for HP*β*CD. Other investigated biomarkers may provide complementary information on neuronal injury, lipid trafficking and treatment mechanisms [21]. Some included biomarkers are PPCS, lysosphingomyelin-509 (Lyso-SM-509), cholestane-triol, heat shock protein 70 (HSP 70), CSF total Tau, fatty acid binding protein 3 (FABP3), calbindin D and plasma glucosylceramides. Future studies should identify their reproducibility, responsiveness to treatment, relationship with age and disease stage, and longitudinal correlation between NPCCSS, SARA, among others. Rather than relying on only one of the two, both biomarkers and clinical endpoints can be combined in order to gain as much information about the disease as possible.

Seizure outcomes were inconsistently evaluated across the included studies, and most classified them as baseline characteristics or AEs as opposed to efficacy outcomes. No conclusion can be made regarding the antiseizure efficacy in NPC due to this. Future trials need to prospectively include seizure parameters like frequency, severity, rescue-medication use, treatment resistance and the effects on both patients’ and caregivers’ QoL. This would help in determining if the interventions are capable of affecting seizures alongside the overall modification of neurological progression.

A relatively recent development in NPC management has been the use of gene therapy in an attempt to counteract the effects of NPC, particularly since it could address underlying NPC1 or NPC2 deficiency [31,41]. There were no eligible interventional human gene therapy trials reported. Research into gene therapy has been predominantly conducted on animal models and should therefore be established as preclinical findings, rather than an applicable finding in human patients [42,43].

There are several limitations involved in the interpretation of this review. NPC is incredibly rare and clinically heterogeneous, which means that sample sizes would be small, with major variations in age, baseline severity, treatment duration, phenotype, and concomitant miglustat exposure. Most non-randomized trials were rated as fair or poor methodological quality, primarily because they lacked concurrent controls, blinded assessments, sample-size justification, or complete follow-up.

Despite these limitations, the evidence supports a meaningful shift from exclusively supportive management to possibly improving neurological function or slowing deterioration. Miglustat had the longest and most consistent evidence for its effects. Arimoclomol provided randomized evidence of slowed progression when used with miglustat, supported by uncontrolled long-term findings. NALL had the clearest randomized evidence of short-term functional improvement, though its potential disease-modifying effects still require long-term confirmation. HP*β*CD demonstrated biological activity, though with inconsistent clinical efficacy and with an SAE of hearing impairment or hearing loss. Efavirenz and nizubaglustat are promising potential strategies for NPC, while vorinostat and NAC still do not have sufficient evidence to support their use. Future multicentre trials should use standardized, clinically meaningful endpoints, prespecified biomarker strategies, concurrent controls where feasible, as well as sufficiently long follow-up to distinguish transient symptomatic improvement from sustained modification of disease progression.

## 5. Conclusions

This systematic review of 24 studies involving approximately 475 unique participants demonstrates that the therapeutic landscape of Niemann–Pick disease type C (NPC) has progressed beyond supportive care, although no available intervention is curative. The therapies proposed for NPC are currently still being studied and examined to ensure that they are being provided to patients in appropriate doses that reflect stabilization or improvement of the disease.

Miglustat has the longest clinical evidence base and appears to stabilize or slow neurological deterioration, particularly when initiated before advanced neurological impairment, but much of its long-term evidence is derived from uncontrolled or observational studies [1,2,3,4,16,32]. Arimoclomol showed promising results in improving neurological symptoms of NPC according to the published trials. When used with miglustat, it reduced disease progression in a randomized trial, with supportive long-term findings from its open-label extension. Its infant substudy primarily provides preliminary safety and pharmacokinetic evidence rather than definitive efficacy. Hypersensitivity reactions and liver-enzyme elevations require monitoring; however, the death reported in the randomized trial was attributed to NPC progression and should not be interpreted as arimoclomol-induced [10,11,17,18]. NALL exhibited the clearest evidence of short-term improvement in ataxia and neurological function and was generally well tolerated. Its open-label extension suggests that these benefits may persist, although the absence of a concurrent comparator limits conclusions regarding long-term disease modification [8,12,19]. HP*β*CD demonstrated biological target engagement and possible stabilization in several uncontrolled intrathecal and intravenous studies, but it caused side effects that were too significant to disregard, namely hearing loss [6,13,20,24,25,26,27]. Efavirenz produced preliminary evidence of cognitive stabilization with a favorable short-term safety profile, but its single-arm design and concomitant miglustat use prevent attribution of the observed outcomes to efavirenz alone [22]. Nizubaglustat demonstrated pharmacodynamic target engagement and acceptable tolerability, but the small mixed-disease population, limited number of participants with NPC, and absence of a long-term placebo comparator mean that its efficacy in NPC remains undetermined [23]. Vorinostat and NAC both demonstrated minimal effect on NPC symptoms, and since only one study was found for each, it is best to research them further before they can be considered as part of the mainstream management of NPC [28,29].

In conclusion, the strongest available evidence supports miglustat, arimoclomol in combination with miglustat, and NALL as treatments capable of slowing neurological deterioration or improving neurological function in selected patients with NPC. However, confidence in the magnitude and durability of these effects remains limited by small samples, overlapping study populations, heterogeneous endpoints, and the predominance of uncontrolled studies. Future multicenter trials should incorporate concurrent comparison groups, standardized clinically meaningful outcomes, validated biomarkers, systematic assessment of seizures and QoL, and sufficiently long follow-up to distinguish short-term symptomatic improvement from sustained modification of disease progression. The hope is that in the future, a cure can be developed for these patients, and these trials and studies are the building blocks that are essential in making that prospective future a reality.

## Figures and Tables

**Figure 1:**
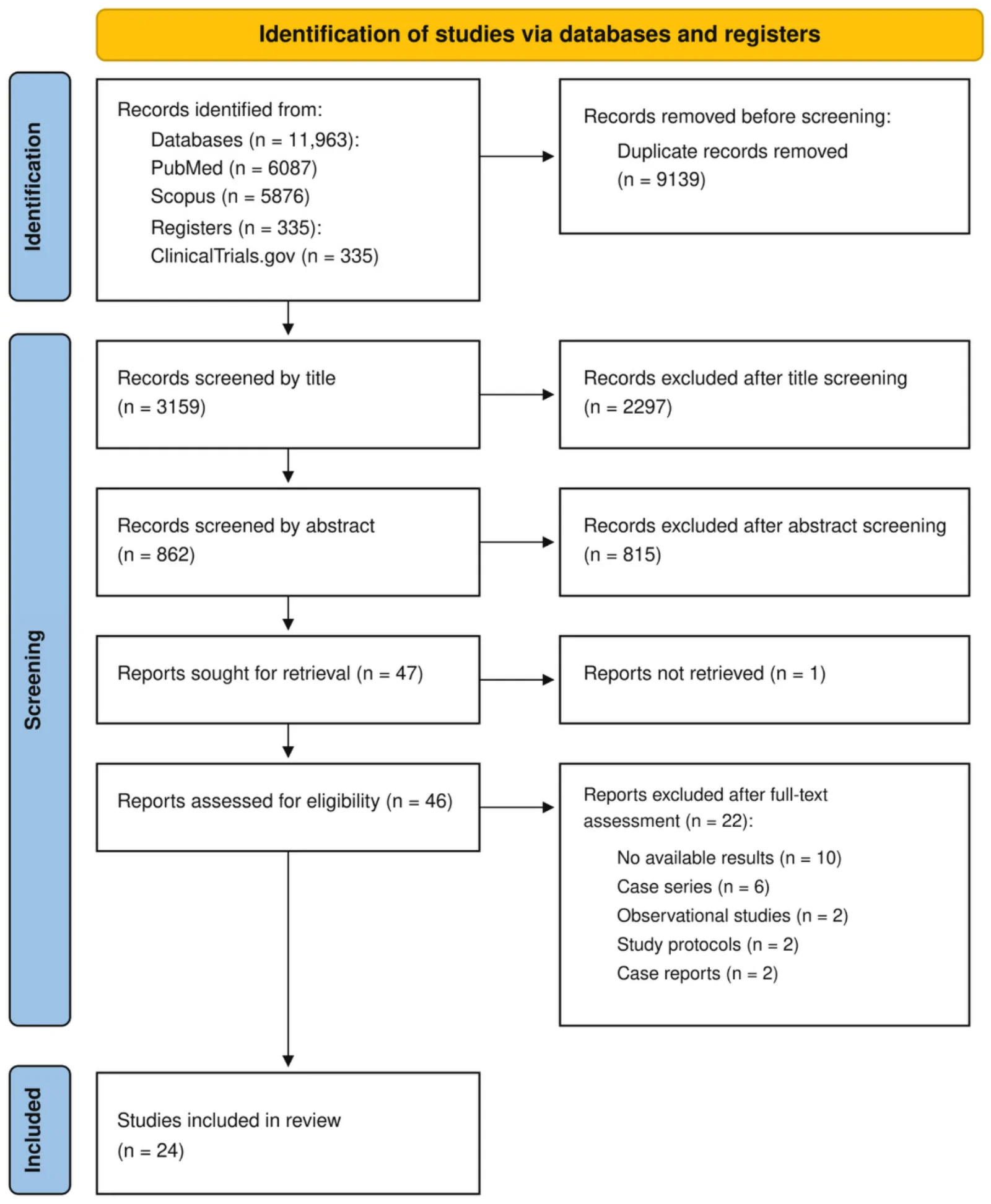
PRISMA flow diagram illustrating the identification, screening, eligibility assessment, and inclusion of studies in the systematic review.

**Figure 2:**
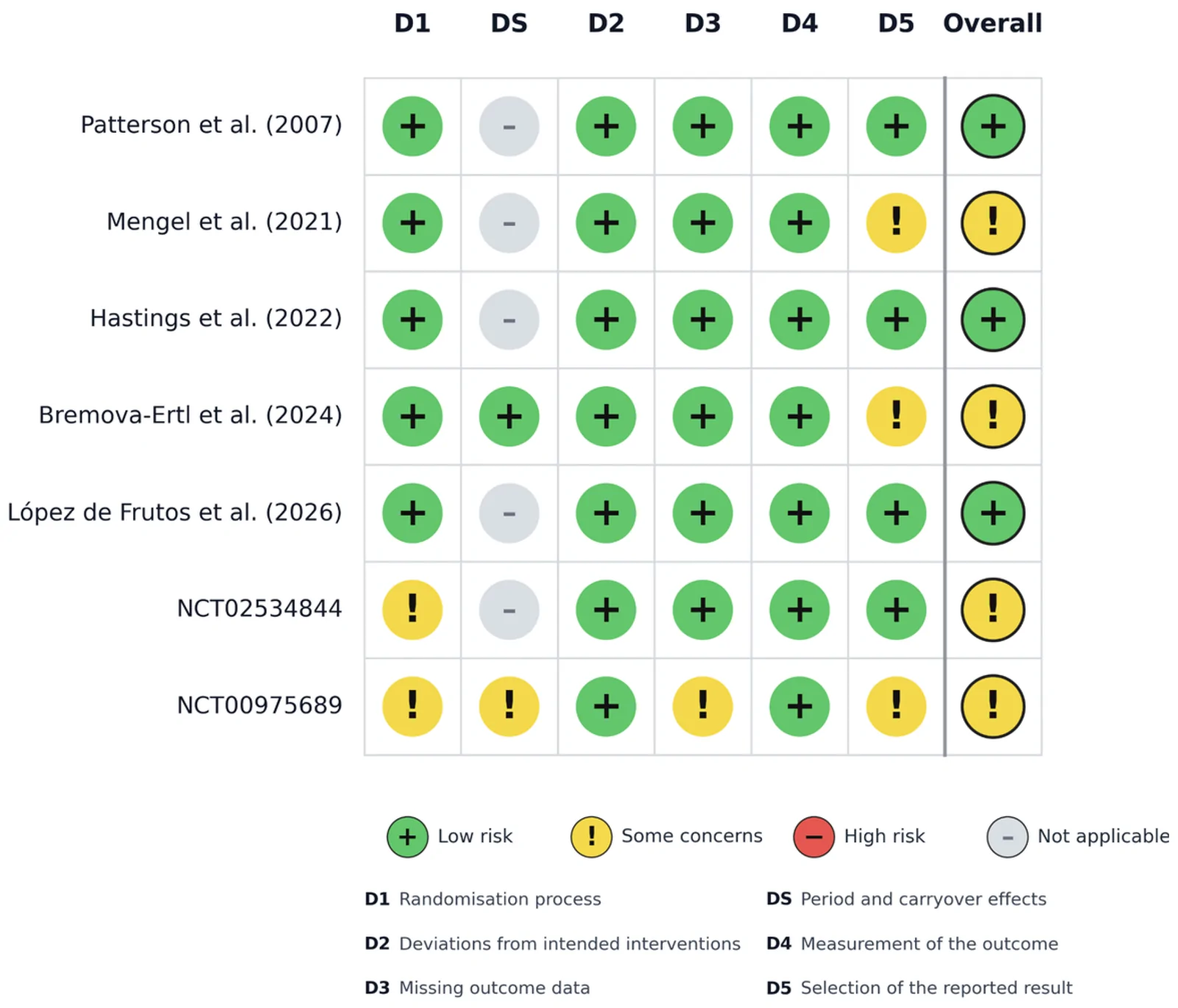
Risk of bias assessment of the included studies using the Cochrane Risk of Bias 2 (RoB 2) tool [6,10,12,16,23,24,29].

## Data Availability

The original contributions presented in this study are included in the article and Supplementary Materials. Further inquiries can be directed to the corresponding author.

## Supplementary Materials

The following supporting information can be downloaded at: https://www.mdpi.com/article/10.3390/biomedicines14102402/s1. Table S1: Detailed eligibility criteria for PubMed and Scopus, Table S2: Detailed eligibility criteria for ClinicalTrials.gov, Table S3: Secondary and exploratory outcomes for PubMed and Scopus, Table S4: Secondary and exploratory outcomes for ClinicalTrials.gov, Table S5: PRISMA 2020 Checklist.
