# Supplementary material for: Therapeutic Interventions in Niemann–Pick Disease Type C: A Systematic Review of Clinical Outcomes and Safety
Source: Biomedicines. 2026 Oct 21;14(10):2402. doi: 10.3390/biomedicines14102402 (PMC13702622; doi:10.3390/biomedicines14102402)
Supplement: Supplementary file 1 [file biomedicines-14-02402-s001.zip › Tables S1-S4.pdf]

Supplementary Table S1. - Detailed eligibility criteria for PubMed and Scopus

S.NO	STUDY TITLE	INCLUSION CRITERIA	EXCLUSION CRITERIA
1.	Miglustat for treatment of Niemann-Pick C disease: a randomized controlled study [16]	Confirmed NPC (↓ cholesterol esterification + abnormal filipin staining); cooperative with physical exam and testing	Clinically significant diarrhea (>3 loose stools/day >7 days, no cause); significant gastrointestinal (GI) disorders; other intercurrent illnesses
2.	Long-Term Miglustat Therapy in Children With Niemann–Pick Disease Type C [3]	Children aged 4–11 years with NPC (confirmed by filipin staining + impaired cholesterol esterification); able to ingest capsule medication.	Significant diarrhea (>3 liquid stools/day for >7 days) within 3 mo; history of major GI disorders; creatinine clearance <70 mL/min/1.73 m^2.
3.	Miglustat in adult and juvenile patients with Niemann–Pick disease type C: Long-term data from a clinical trial [2]	Male and female patients aged ≥12 years with NPC (confirmed by abnormal cholesterol esterification + filipin staining in cultured fibroblasts).	Not mentioned
4.	Miglustat therapy in the French cohort of paediatric patients with Niemann-Pick disease type C [4]	Confirmed NPC diagnosis via filipin staining and/or genetic testing	Not mentioned
5.	Long term follow-up to evaluate the efficacy of miglustat treatment in Italian patients with Niemann-Pick disease type C [1]	Diagnosed with NPC (biochemical tests: filipin staining, LDL-cholesterol esterification; molecular analysis: NPC1/NPC2 genes)	Not mentioned
6.	Efficacy and safety of arimoclomol in Niemann-Pick disease type C: Results from a double-blind, randomised, placebo-controlled, multinational phase 2/3 trial of a novel treatment. [10]	Age 2–18 years; confirmed NPC1/NPC2; ≥1 neurological sign; able to walk independently or with assistance; stable miglustat for ≥6 months, where applicable	Concurrent interventional trial; severe hepatic or renal insufficiency; recent investigational drug; asymptomatic neurological disease; uncontrolled seizures; absence of consent
7.	Arimoclomol in infants with Niemann-Pick disease type C: Results from the phase 2/3 open-label pediatric substudy [18]	Age 6 to <24 months; confirmed NPC1/NPC2; stable miglustat for ≥1 month, where applicable	Transaminases >3 times ULN; renal insufficiency; known active liver disease; prolonged icterus; organ malformations; previous liver transplantation; clinical condition preventing protocol-required blood collection.
8.	Long-term efficacy and safety of arimoclomol in Niemann-Pick disease type C: Final results of the phase 2/3 NPC-002 48-month open-label extension trial [17]	Completed the double-blind phase; received ≥1 open-label arimoclomol dose; investigator considered continued treatment beneficial	Original exclusions included severe hepatic or renal insufficiency, neurologically asymptomatic disease, and uncontrolled seizures

9.	Efficacy and safety of N-acetyl-L-leucine in Niemann-Pick disease type C. [8]	Confirmed NPC1/NPC2; age ≥6 years in Europe or ≥18 years in the United States; Scale for the Assessment and Rating of Ataxia (SARA) score 5–33; weight ≥15 kg; stable permitted therapy	Asymptomatic or genetically unconfirmed NPC; chronic diarrhea; malignancy; insulin-dependent diabetes; relevant hypersensitivity or comorbidity; abnormal liver tests; recent investigational treatment; pregnancy or breastfeeding; inability to discontinue prohibited medication
10.	Trial of N-Acetyl-l-Leucine in Niemann-Pick Disease Type C [12]	Age ≥4 years; genetically confirmed symptomatic NPC; SARA score 7–34; weight ≥15 kg; stable permitted treatment; 42-day washout from prohibited medication	Relevant hypersensitivity; recent investigational drug; condition affecting safety or assessment reliability; substance misuse; pregnancy or breastfeeding; severe sensory or musculoskeletal impairment; inability to discontinue prohibited medication
11.	Disease-Modifying, Neuroprotective Effect of N-Acetyl- l -Leucine in Adult and Pediatric Patients with Niemann-Pick Disease Type C [19]	Completed the parent trial; investigator considered continued NALL potentially beneficial; written consent	Parent-trial exclusions included age <4 years, asymptomatic disease, and inability to complete functional assessments reliably
12.	Intrathecal 2-hydroxypropyl-β-cyclodextrin decreases neurological disease progression in Niemann-Pick Disease, type C1: an ad-hoc analysis of a non-randomized, open-label, phase 1/2 trial [13]	Confirmed symptomatic NPC1; eligibility age 2–25 years; weight >12 kg; stable miglustat, where applicable; able to undergo repeated blood and cerebrospinal fluid (CSF) collection	Severe or asymptomatic disease; prior cyclodextrin treatment; relevant hypersensitivity, infection, organ dysfunction, unstable seizures, or lumbar-puncture contraindication; pregnancy or breastfeeding
13.	Long-Term Neuropsychological Outcomes from an Open-Label Phase I/IIa Trial of 2-Hydroxypropyl-β-Cyclodextrins (VTS-270) in Niemann-Pick Disease, Type C1. [20]	Neurologically symptomatic NPC1; eligibility age 2–25 years; able to complete repeated assessments	Severe disease preventing participation; prior cyclodextrin treatment; major infection or organ dysfunction; unstable seizures; lumbar-puncture contraindication; pregnancy or breastfeeding
14.	Intravenous 2-hydroxypropyl-β-cyclodextrin (Trappsol® Cyclo™) demonstrates biological activity and impacts cholesterol metabolism in the central nervous system and peripheral tissues in adult subjects with Niemann-Pick Disease Type C1: Results of a phase 1 trial. [6]	Age ≥18 years; confirmed NPC1; 17-domain NPC-CSS ≤30; ≥1 systemic manifestation; stable miglustat permitted	NPC2; recent cyclodextrin or investigational treatment; relevant organ dysfunction; weight <40 or >100 kg; inability to comply or provide consent; pregnancy risk
15.	Long-term administration of intravenous Trappsol® Cyclo™ (HP-β-CD) results in clinical benefits and stabilization or slowing of disease progression in patients with Niemann-Pick disease type C1: Results of an international 48-week Phase I/II trial [21]	Age ≥2 years; confirmed NPC1 based on two NPC1 variants, one variant plus positive filipin staining, or vertical supranuclear gaze palsy plus an NPC1 variant/positive filipin staining and no NPC2 variant; 17-Domain Niemann-Pick Disease Type C-Clinical Severity Scale (17D-NPC-CSS) ≤30, with no more than four major	NPC2 variants; stage 3 chronic kidney disease; acute liver disease; or body weight >100 kg.

		domains scoring ≥3; negative pregnancy test where applicable; informed consent.	
16.	Efficacy and safety of efavirenz in Niemann-Pick disease type C [22]	Age ≥14 years; genetically confirmed late-juvenile/adult-onset NPC; Clinical Dementia Rating (CDR) 0.5–2 or CDR-Sum of Boxes (CDR-SB) ≤12; ≥8 years of education; receiving miglustat	Chronic liver disease; unstable epilepsy
17.	The RAINBOW study: a phase 2 trial evaluating the 3-month pharmacokinetic and pharmacodynamic data and 18-month clinical and safety outcomes of nizubaglustat in GM2 gangliosidoses or Niemann–Pick type C disease [23]	Age ≥12 years; genetically confirmed GM2 or NPC; Scale for the Assessment and Rating of Ataxia (SARA) score ≥1; able to complete assessments; NPC participants unable or unwilling to take miglustat	Confounding medical or neurological condition; unstable antiseizure therapy; significant hepatic, renal, hematological, or cardiac abnormality; recent investigational or prohibited treatment; pregnancy or breastfeeding; relevant hypersensitivity or suicidal intent

Supplementary Table S2. - Detailed eligibility criteria for ClinicalTrials.gov.

S.NO	STUDY TITLE	INCLUSION CRITERIA	EXCLUSION CRITERIA
1.	A Phase 2b/3 Prospective, Randomized, Double-Blind, Sham-Controlled 3-Part Trial of VTS-270 (2-hydroxypropyl-β-cyclodextrin) in Subjects With Neurologic Manifestations of Niemann-Pick Type C1 (NPC1) Disease [24]	Confirmed symptomatic NPC1; neurological onset before age 15; eligible severity score; able to undergo lumbar puncture; stable miglustat and seizure treatment	Severe disease; weight <15 kg; previous intrathecal HPβCD; relevant hypersensitivity, spinal disease, infection, laboratory abnormality, uncontrolled seizures, hydrocephalus, bleeding risk, or non-compliance
2.	A Phase 2b/3 Prospective, Randomized, Double-blind, Sham-controlled Trial of VTS-270 (2-hydroxypropyl-β-cyclodextrin) in Subjects with Neurologic Manifestations of Niemann-Pick Type C1 (NPC1) Disease [25]	Conversion from the NIH monthly regimen, sponsor-authorized amended dosing, or direct authorization for Part C enrollment	Failure to meet an authorized Part C entry pathway
3.	A Phase 2b/3 Open-label Trial of VTS-270 (2-hydroxypropyl-β-cyclodextrin) in Subjects With Neurologic Manifestations of Niemann-Pick Type C1 Disease Previously Treated Under Protocol VTS301 [26]	Completed or received rescue treatment in VTS301 Part B and continued into Part C; investigator supported continued therapy; consent/assent	Previous discontinuation for an AE; unresolved serious AE; pregnancy or breastfeeding; infection; spinal or lumbar disease; significant laboratory abnormality; hydrocephalus; anticoagulant use; active pulmonary disease; unacceptable risk

4.	Open-label Evaluation of Adrabetadex in Patients with Neurologic Manifestations of Niemann-Pick Type C Disease (NPC) [27]	Age ≥4 years; confirmed neurologically symptomatic NPC; able to undergo lumbar puncture; stable miglustat and seizure treatment; responsible caregiver available	Weight <15 kg; relevant hypersensitivity; recent investigational product; pregnancy or breastfeeding; infection or psychosis; bleeding risk; significant laboratory abnormality; spinal disease; hydrocephalus; non-compliance or unacceptable risk
5.	Phase 1/2 Study of Vorinostat Therapy in Niemann-Pick Disease, Type C1 [28]	Confirmed symptomatic NPC1; age 18–60 years; qualifying fibroblast response to vorinostat; stable miglustat; able to attend NIH and undergo blood and cerebrospinal fluid (CSF) sampling	Severe disease; previous cyclodextrin or histone deacetylase inhibitor (HDACi); relevant hypersensitivity, infection, laboratory abnormality, pulmonary disease, uncontrolled seizures, thromboembolism, or pregnancy
6.	Biomarker Validation for Niemann-Pick Disease, Type C: Safety and Efficacy of N-Acetyl Cysteine [29]	Confirmed NPC; age ≥12 months; weight >10 kg; able to receive oral medication orally or through a gastrostomy tube	Unable to travel or tolerate procedures; pregnancy; NAC hypersensitivity; prohibited medication; uncontrolled seizures; unstable miglustat; gastrointestinal bleeding risk; sodium restriction; significant hepatic, hematological, or renal abnormality
7.	Evaluation of the safety and efficacy of miglustat for the treatment of Chinese patients with Niemann-Pick disease type C: A prospective, open-label, single-arm, phase IV trial [30]	Age ≥4 years; confirmed NPC1/NPC2; able to complete saccadic-eye-movement testing and swallow treatment; appropriate consent and contraception	Significant diarrhea; treatment hypersensitivity; severe renal impairment; pregnancy or breastfeeding; prohibited treatment; end-stage disease; another relevant neurological disorder; uncontrolled epilepsy; complete ophthalmoplegia; life expectancy <12 months

Supplementary Table S3. - Secondary and exploratory outcomes for PubMed and Scopus

S.NO	STUDY TITLE	SECONDARY OUTCOMES
1.	Miglustat for treatment of Niemann-Pick C disease: a randomized controlled study [16]	Horizontal saccadic eye movement initial peak velocity–amplitude slope (HSEM-β); swallowing; auditory acuity; ambulation; Mini-Mental State Examination (MMSE) in participants aged ≥12 years
2.	Long-Term Miglustat Therapy in Children With Niemann–Pick Disease Type C [3]	HSEM-β; vertical saccadic parameters; neurological and neuropsychological assessments; swallowing; ambulation
3.	Miglustat in adult and juvenile patients with Niemann–Pick disease type C: Long-term data from a clinical trial [2]	HSEM-β, swallowing, ambulation, cognition using MMSE

4.	Miglustat therapy in the French cohort of paediatric patients with Niemann-Pick disease type C [4]	Seizure control, cataplexy status, psychometric performance, hearing function, brainstem auditory evoked potentials (BAEP), MRI findings, MRS results (NAA, Cho, Cho/NAA), peripheral neuropathy status by electromyography (EMG), swallowing ability (gastrostomy/NG tube)
5.	Long term follow-up to evaluate the efficacy of miglustat treatment in Italian patients with Niemann-Pick disease type C [1]	Safety; physical and biochemical changes; videofluoroscopic swallowing assessment in four children
6.	Efficacy and safety of arimoclomol in Niemann-Pick disease type C: Results from a double-blind, randomised, placebo-controlled, multinational phase 2/3 trial of a novel treatment [10]	Clinical Global Impression of Improvement (CGI-I); NPCCSS responders and worsening; 17-domain NPCCSS; Scale for the Assessment and Rating of Ataxia (SARA); nine-hole peg test (9-HPT); EuroQol five-dimension questionnaire (EQ-5D)
7.	Arimoclomol in infants with Niemann-Pick disease type C: Results from the phase 2/3 open-label pediatric substudy [18]	Bayley Scales of Infant and Toddler Development, Third Edition (BSID-III); pharmacokinetics; cholestane-triol and heat-shock protein 70 (HSP70)
8.	Long-term efficacy and safety of arimoclomol in Niemann-Pick disease type C: Final results of the phase 2/3 NPC-002 48-month open-label extension trial [17]	Seventeen-domain NPCCSS; NPC clinical database (NPC-cdb) score; long-term safety
9.	Efficacy and safety of N-acetyl-L-leucine in Niemann-Pick disease type C. [8]	SARA total score (ataxia);(modified disability rating scale mDRS) (6-domain disability); SCAFI (Spinocerebellar Ataxia Functional Index)(8-meter walk test (8MWT), 9HPT, PATA); clinical global impression of severity (CGI-S)/Clinical Global Impression of Change (CGI-C) (investigator, caregiver, patient); EQ-5D (QoL)
10.	Trial of N-Acetyl-l-Leucine in Niemann-Pick Disease Type C. [12]	mDRS; SCAFI; CGI-I; EQ-5D-5L (5-level) (adult QoL); EQ-5D-Y (child visual analogue QoL); exit interviews
11.	Disease-Modifying, Neuroprotective Effect of N-Acetyl- l -Leucine in Adult and Pediatric Patients with Niemann-Pick Disease Type C [19]	15-domain NPC-CSS, 4-domain NPC-CSS and SARA.Prespecified analyses by paediatric/adult status and background miglustat use.
12.	Intrathecal 2-hydroxypropyl-β-cyclodextrin decreases neurological disease progression in Niemann-Pick Disease, type C1: an ad-hoc analysis of a non-randomized, open-label, phase 1/2 trial [13]	Clinical efficacy progression via NPC Neurological Severity Score (NSS), including total NNSS, NNSS excluding hearing and individual domains.CSF 24(S)-HC, FABP3 and calbindin D biomarkers; audiological safety; responder analysis.
13.	Long-Term Neuropsychological Outcomes from an Open-Label Phase I/IIa Trial of 2-Hydroxypropyl-β-Cyclodextrins (VTS-270) in Niemann-Pick Disease, Type C1. [20]	Memory (immediate, delayed, recognition) – wide range assessment of memory & learning, second addition (WRAML-2)
14.	Intravenous 2-hydroxypropyl-β-cyclodextrin (Trappsol® Cyclo™) demonstrates biological activity and impacts cholesterol metabolism in the central nervous system and peripheral tissues in adult subjects with Niemann-Pick Disease Type C1: Results	PD biomarkers (peripheral and central nervous system (CNS)), Clinical severity (17D-NPC-CSS), Exploratory quality-of-life observations

	of a phase 1 trial. [6]	
15.	Long-term administration of intravenous Trappsol® Cyclo™ (HP-β-CD) results in clinical benefits and stabilization or slowing of disease progression in patients with Niemann-Pick disease type C1: Results of an international 48-week Phase I/II trial [21]	Plasma and cerebrospinal fluid (CSF) pharmacokinetics (PK); cholesterol-metabolism biomarkers, including 24S-hydroxycholesterol (24S-HC) and N-palmitoyl-O-phosphocholineserine (PPCS); liver and spleen size; Scale for the Assessment and Rating of Ataxia (SARA); Clinical Global Impression of Improvement (CGI-I); Patient Global Impression of Change (PGIC); bead-threading; Pediatric Quality of Life survey (PedsQL); safety and audiometry.
16.	Efficacy and safety of efavirenz in Niemann-Pick disease type C [22]	Neuropsychological: Individual scores from the instruments included in the primary composite, together with global CDR, BNT, JLO, DEX, NPI, AES and BDI.Neurological: Changes from baseline through Week 52 in SARA, EAT-10, PDS and the WAIS-III block-design and symbol-search subtests.Biological: Brain imaging, CSF proteins and plasma 24-hydroxycholesterol.Safety: Cumulative incidence and seriousness of adverse events.
17.	The RAINBOW study: a phase 2 trial evaluating the 3-month pharmacokinetic and pharmacodynamic data and 18-month clinical and safety outcomes of nizubaglustat in GM2 gangliosidoses or Niemann–Pick type C disease [23]	Changes from baseline during the extension in SARA, functional SARA (fSARA) and Inventory of Non-Ataxia Signs (INAS) count; long-term safety and tolerability; and seizure frequency recorded using participant or caregiver diaries.

Supplementary Table S4. - Secondary and exploratory outcomes for ClinicalTrials.gov.

S.NO	STUDY TITLE	SECONDARY OUTCOMES
1.	A Phase 2b/3 Prospective, Randomized, Double-Blind, Sham-Controlled 3-Part Trial of VTS-270 (2-hydroxypropyl-β-cyclodextrin) in Subjects With Neurologic Manifestations of Niemann-Pick Type C1 (NPC1) Disease [24]	NPC-SS total excluding hearing/ABR; CGIC responders; NPC-SS responders; EQ-5D-3L VAS; rescue qualification; nine NPC-SS domains; total NPC-SS including hearing/ABR; time to one-point composite worsening; TUG; nine-hole peg test; TEAEs; annualized NPC-SS slope
2.	Phase 1/2 Study of Vorinostat Therapy in Niemann-Pick Disease, Type C1 [28]	Serum cathepsin D and galectin-3 concentrations at baseline and 6 months
3.	Evaluation of the safety and efficacy of miglustat for the treatment of Chinese patients with Niemann-Pick disease type C: A prospective, open-label, single-arm, phase IV trial [30]	Change in Pineda Disability Scale scores; safety and tolerability; height and body weight among paediatric participants
